# Harmonization of clinical practice guidelines for primary prevention and screening: actionable recommendations and resources for primary care

**DOI:** 10.1186/s12875-024-02388-3

**Published:** 2024-05-06

**Authors:** Carolina Fernandes, Denise Campbell-Scherer, Aisha Lofters, Eva Grunfeld, Kris Aubrey-Bassler, Heidi Cheung, Katherine Latko, Wendy Tink, Richard Lewanczuk, Melissa Shea-Budgell, Ruth Heisey, Tracy Wong, Huiming Yang, Sakina Walji, Margo Wilson, Elizabeth Holmes, Kelly Lang-Robertson, Christina DeLonghi, Donna Patricia Manca

**Affiliations:** 1 Department of Family Medicine, University of Alberta, Edmonton, AB Canada; 2 Office of Lifelong Learning and the Physician Learning Program, Faculty of Medicine and Dentistry, University of Alberta, Edmonton, AB Canada; 3 Department of Family and Community Medicine, University of Toronto, Toronto, ON Canada; 4 Peter Gilgan Centre for Women’s Cancers, Women’s College Hospital, Toronto, ON Canada; 5 Ontario Institute for Cancer Research, Toronto, ON Canada; 6 Discipline of Family Medicine, Memorial University of Newfoundland, St. John’s, NL Canada; 7 Newfoundland and Labrador Centre for Health Information, St. John’s, NL Canada; 8 College of Physicians and Surgeons of Ontario, Toronto, ON Canada; 9 Department of Family Medicine, University of Calgary, Calgary, AB Canada; 10 Alberta Health Services, Alberta, AB Canada; 11 Department of Medicine, University of Alberta, Edmonton, AB Canada; 12 Arnie Charbonneau Cancer Institute, University of Calgary, Calgary, AB Canada; 13 Family and Community Medicine, Women’s College Hospital, Toronto, ON Canada; 14 Strategic Clinical Networks, Alberta Health Services, Calgary, AB Canada; 15 Department of Family Medicine, Mount Sinai Hospital, Sinai Health System, Toronto, ON Canada; 16 Discipline of Emergency Medicine, Memorial University of Newfoundland, St. John’s, NL Canada; 17 Canadian Cancer Society, Toronto, ON Canada; 18 Centre for Effective Practice, Toronto, ON Canada

**Keywords:** Clinical practice guidelines, Prevention, Primary care, Screening, Chronic disease

## Abstract

**Background:**

Clinical practice guidelines (CPGs) synthesize high-quality information to support evidence-based clinical practice. In primary care, numerous CPGs must be integrated to address the needs of patients with multiple risks and conditions. The BETTER program aims to improve prevention and screening for cancer and chronic disease in primary care by synthesizing CPGs into integrated, actionable recommendations. We describe the process used to harmonize high-quality cancer and chronic disease prevention and screening (CCDPS) CPGs to update the BETTER program.

**Methods:**

A review of CPG databases, repositories, and grey literature was conducted to identify international and Canadian (national and provincial) CPGs for CCDPS in adults 40–69 years of age across 19 topic areas: cancers, cardiovascular disease, chronic obstructive pulmonary disease, diabetes, hepatitis C, obesity, osteoporosis, depression, and associated risk factors (i.e., diet, physical activity, alcohol, cannabis, drug, tobacco, and vaping/e-cigarette use). CPGs published in English between 2016 and 2021, applicable to adults, and containing CCDPS recommendations were included. Guideline quality was assessed using the Appraisal of Guidelines for Research and Evaluation (AGREE) II tool and a three-step process involving patients, health policy, content experts, primary care providers, and researchers was used to identify and synthesize recommendations.

**Results:**

We identified 51 international and Canadian CPGs and 22 guidelines developed by provincial organizations that provided relevant CCDPS recommendations. Clinical recommendations were extracted and reviewed for inclusion using the following criteria: 1) pertinence to primary prevention and screening, 2) relevance to adults ages 40–69, and 3) applicability to diverse primary care settings. Recommendations were synthesized and integrated into the BETTER toolkit alongside resources to support shared decision-making and care paths for the BETTER program.

**Conclusions:**

Comprehensive care requires the ability to address a person’s overall health. An approach to identify high-quality clinical guidance to comprehensively address CCDPS is described. The process used to synthesize and harmonize implementable clinical recommendations may be useful to others wanting to integrate evidence across broad content areas to provide comprehensive care. The BETTER toolkit provides resources that clearly and succinctly present a breadth of clinical evidence that providers can use to assist with implementing CCDPS guidance in primary care.

**Supplementary Information:**

The online version contains supplementary material available at 10.1186/s12875-024-02388-3.

## Background

Turning the tide on chronic disease is a major health system priority and integral to improving health outcomes, services, and costs. Seventy-three percent of Canadians 65 years of age and older have at least 1 of the 10 most common cancers and/or chronic diseases [1]. Many of these could be prevented through management of modifiable risk factors and early detection through screening [2].

Primary care provides the first contact for patients in the healthcare system and therefore is an ideal setting to implement cancer and chronic disease prevention and screening (CCDPS) in Canada. Unfortunately, a substantial gap exists between clinical recommendations for CCDPS and actual practice [2–6]. Due to a fragmented healthcare system and provider time constraints, implementation of guidelines tends to focus on recommendations for specific organ systems, medical conditions, or single risk factors [6, 7]. Initiatives such as the Canadian Cardiovascular Harmonized National Guidelines Endeavour (C-CHANGE) [8–10] and the Building on Existing Tools to Improve Chronic Disease Prevention and Screening in Primary Care (BETTER) program [6, 11] exemplify efforts to present compilations of high-quality, evidence-based recommendations across multiple conditions in a way that is accessible and actionable by primary care providers (PCPs).

The BETTER program [12], with over 15 years of in-depth study, has developed a novel, comprehensive approach to CCDPS in primary care based on the Chronic Care Model (CCM), which identifies essential elements needed to provide comprehensive, proactive care for patients with chronic conditions from health promotion to disease management [13–15]. This approach introduces an enhanced role, the Prevention Practitioner (PP), typically undertaken by a clinician not responsible for ongoing care decisions or routine care. The PP works directly with patients to determine which CCDPS actions they are eligible to receive, and through a process involving shared decision-making and S.M.A.R.T. (specific, measurable, attainable, realistic, time-bound) goal setting, develops a unique, personalized “prevention prescription” with each patient [16]. The evidence-based prevention prescription is rooted in harmonized, high-quality CCDPS guidelines and tailored to patients based on their medical history, risk factors, and family history. This cost-effective intervention has been demonstrated to improve uptake of CCDPS actions in urban primary care settings as compared to usual care [2] and similar improvements have been observed in rural and remote communities [17] and public health settings [18] across Canada.

In this paper, we describe the rigorous process undertaken to synthesize and harmonize high-quality CCDPS clinical practice guidelines (CPGs) to update the clinical recommendations used in the BETTER program. This work was part of the Building on Existing Tools to Improve Cancer and Chronic Disease Prevention and Screening in Primary Care for Wellness of Cancer Survivors and Patients (BETTER WISE) project, a multi-provincial cluster randomized controlled trial (cRCT) [19]. The results from the BETTER WISE trial, including patient-level outcomes to assess the effectiveness of the approach and qualitative findings describing the impacts of the global pandemic of coronavirus disease 2019 (COVID-19) on the trial and overall prevention and screening services in primary care, are reported elsewhere [20–22]. We also describe the updated BETTER toolkit, a unique set of resources and tools aimed at supporting prevention of multiple cancers and chronic conditions in the primary care setting. Refinement of the toolkit was undertaken due to changes to the clinical evidence since its last iteration and to include new content areas informed by emerging evidence and feedback received from end-users. The BETTER toolkit informs the PP role and provides PCPs with accessible evidence-based clinical practice tools to address CCDPS.

## Methods

### Overview of the evidence review and CPG harmonization process

The process used to search, identify, appraise, synthesize, and harmonize CPGs for the BETTER program builds on our previous work [6, 11], and involved the development of a compilation of robust CCDPS recommendations across the targeted conditions and risk factors while ensuring these were actionable and implementable in diverse settings across Canada. The clinical recommendations previously included were appliable to patients 40–65 years of age and encompassed the following areas: 1) cancers—breast, cervical, colorectal, lung and prostate; 2) chronic diseases – diabetes, cardiovascular disease, and obesity; 3) other conditions – depression; and 4) lifestyle risk factors – diet, physical activity, and alcohol and tobacco use. As described elsewhere [6, 11], implementation science theories, models, and frameworks were used to inform the BETTER program and the approach undertaken to update the clinical evidence for the program to ensure that these recommendations were relevant, practical, feasible, and implementable in primary care settings.

The BETTER evidence review and CPG synthesis/harmonization process involved 3 main phases: 1) evidence review and identification of high-quality CPGs; 2) guideline synthesis and harmonization to standardize recommendations; and 3) refinement of the BETTER toolkit. These 3 phases were conceptualized within the Canadian Institutes of Health Research (CIHR) knowledge-to-action process model, a conceptual framework that depicts the process of knowledge translation as a continuous cycle with knowledge creation at the core and the activities related to “action” or application/implementation of created knowledge at the periphery [6, 11, 19, 23]. This model has been identified as an implementation tool that can guide the process of translating evidence into practice [24]. This process culminated in the refinement and creation of knowledge resources and tools to support primary prevention and screening for relevant cancers and chronic diseases: the BETTER toolkit. Composed of clinical tools that succinctly and visually represent the clinical recommendations used in the program as well as a collection of accessible programs and resources available to providers and patients to support CCDPS efforts, the BETTER toolkit includes: a health survey, care paths for prevention and screening, a prevention prescription, a S.M.A.R.T. goals sheet, and bubble diagrams which provide CCDPS targets for patients at average risk.

### Phase 1: evidence review and identification of high-quality CPGs

#### Review of the literature

An evidence review involving a targeted search strategy developed by the Centre for Effective Practice (CEP) [25] (a non-profit independent knowledge translation organization based in Toronto, Canada,) was conducted for 19 topics related to CCDPS in the following areas: 1) cancers—breast, cervical, colorectal, lung and prostate; 2) chronic diseases – chronic obstructive pulmonary disease (COPD), diabetes, cardiovascular disease, hepatitis C, obesity, and osteoporosis/bone health; 3) other conditions – depression; and 4) lifestyle risk factors – diet, physical activity, alcohol, cannabis, drug, tobacco, and vaping/e-cigarette use.

#### Search criteria and search strategy

The search updated the previous evidence review, which was conducted in 2016 [6, 11], and was limited to CPGs published in English between 2016 and 2021 focused on adults. Priority was given to Canadian guidelines (national and provincial – Alberta, Ontario, Newfoundland and Labrador, and Nova Scotia as these jurisdictions were engaged in the BETTER WISE project or actively implementing the BETTER program). Guidelines were excluded if they solely provided recommendations for diagnosis, treatment, or management of cancer, and not screening or prevention of cancer, or if they were not applicable to the primary care setting as the focus of the BETTER program is primary prevention and screening of cancer and chronic disease in primary care.

The 4-step search strategy used by the CEP to identify CPGs for each topic is described in Table 1. Specific details regarding the search strategies for each topic are provided in Appendix 1. Titles and abstracts of CPGs identified through this search were reviewed for relevance, and promising results were retrieved in full-text for further consideration.

**Table 1:** Four-step search process for high-quality clinical practice guidelines

1) Topic-specific Databases: • Ontario Health: Cancer Care Ontario: https://www.cancercareontario.ca/en/guidelines-advice Canadian Partnership Against Cancer Guidelines Database^a^: https://www.partnershipagainstcancer.ca/work-with-us/procurement/procurement-bid/cancer-guidelines-database/
2) Clinical Practice Guideline Databases: • ECRI Guidelines Trust: https://www.ecri.org/solutions/ecri-guidelines-trust • Canadian Medical Association (CMA) CPG Infobase: https://joulecma.ca/cpg/homepage
3) Key Guideline Developers: • Canadian Task Force on Preventive Health Care: https://canadiantaskforce.ca/guidelines/published-guidelines/ • National Institute for Health and Care Excellence: http://www.nice.org.uk/ • Scottish Intercollegiate Guidelines Network: https://www.sign.ac.uk/our-guidelines/ • Toward Optimized Practice (TOP) Practice Guidelines: https://actt.albertadoctors.org/cpgs/ • U.S. Preventative Services Task Force: http://www.ahrq.gov/clinic/prevnew.htm • Province Specific Bodies - Alberta Health Services - Eastern Health - Health Quality Ontario - Nova Scotia Health - Ontario Health
4) Grey Literature via a General Internet Search Google: ([Topic terms] AND [“Guideline” or “Screening guideline”]). First 3 pages of results examined

^a^The Canadian Partnership Against Cancer has ceased funding for the Cancer Guidelines Database and it is no longer available online

#### CPG quality appraisal

Full-text guidelines underwent an initial quality appraisal using the Appraisal of Guidelines for Research and Evaluation (AGREE) II Instrument, the purpose of which is to evaluate the process of CPG development and quality of reporting [26]. To pass the initial quality check performed by trained reviewers at the CEP, a guideline had to include clear recommendations linked to levels of evidence and be based on a systematic review of the literature (items 7 and 12 in the “Rigour of Development” domain of AGREE II). CPGs that fulfilled these criteria were appraised independently by 2 reviewers using the full AGREE II, consisting of 23 items across 6 domains and an overall quality score, in preparation for Phase 2. These appraisals were subsequently reviewed by a lead expert reviewer and reconciled into a single score to provide an overall summary of the methodological quality of each guideline.

A review was also conducted to identify guidelines published by provincial organizations in the 4 jurisdictions of interest. Provincial guidance documents published between 2016 and 2021, regardless of whether or not they passed the “Rigour of Development” criteria described above, were included for review in Phase 2 because of their relevance to the local context, as the BETTER program was actively being implemented in these jurisdictions and we needed to ensure that the recommendations incorporated into the program would be actionable, accounting for regional differences. The recommendations from the included high-quality CPGs and key provincial guidelines were extracted into evidence tables for review in Phase 2, which also included each recommendation’s level of evidence and the strength of recommendation, where available.

### Phase 2: guideline synthesis and harmonization to standardize recommendations

**Fig. 1:**
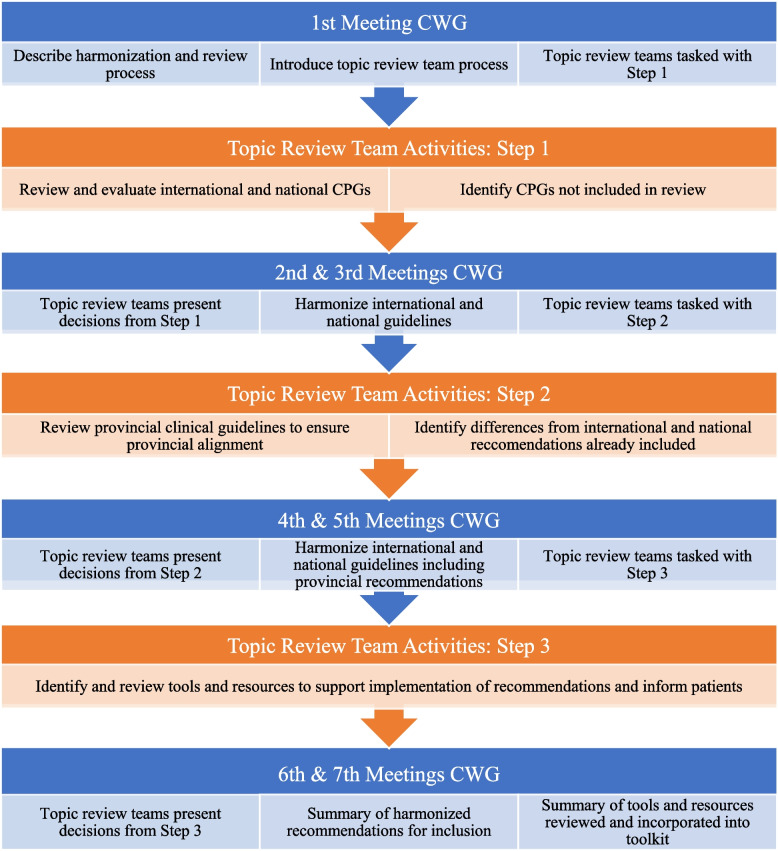
Phase 2: guideline harmonization process

The BETTER Clinical Working Group (CWG) was convened for 5 months (January to May 2022) to review the international and Canadian CPGs identified through Phase 1 and to integrate recommendations and resources for inclusion in the BETTER program (see Fig. 1). Members of the CWG included patients, health policy experts (regional, provincial, federal; government and non-government), PCPs, content experts (e.g., cancer, obesity, diabetes, lifestyle risk factors), and primary care and primary healthcare researchers who shared their perspectives to synthesize and harmonize the identified CPGs into clear, actionable recommendations. In this phase, the CWG was divided into 3 topic-review teams based on their area(s) of expertise:

1. Cancer team (5 members), focusing on breast, cervical, colorectal, lung, and prostate cancer;
2. Chronic disease team (10 members), focusing on cardiovascular disease, COPD, depression, diabetes, hepatitis C, obesity, and osteoporosis; and
3. Lifestyle risk factor team (4 members), focusing on alcohol, cannabis, drug, tobacco, and vaping/e-cigarette use, diet, and physical activity.

Topic-review teams worked independently to evaluate each recommendation within their ambit for clarity, actionability, and to determine if the recommendation could be operationalized in the primary care setting based on the following criteria: 1) focus on primary prevention and screening; 2) relevance to adults 40–69 years of age, the age group to which most CCDPS recommendations apply; and 3) applicability to primary care settings across different Canadian jurisdictions. Specifically, team members voted “yes” (i.e., include in BETTER), “no” (i.e., do not include in BETTER) with rationale for any “no” votes clearly documented, and “maybe” (i.e., further discussion required). The topic-review teams presented their assessments to the larger CWG at scheduled meetings. Any disagreements or ambiguities were resolved through discussion until consensus for inclusion or exclusion was reached. In cases where multiple individual recommendations for a topic were endorsed for inclusion, these were combined, harmonized, and simplified when appropriate, through group discussion. Recommendations emerging from provincial guidance documents that did not meet the initial quality appraisal criteria were reviewed and harmonized alongside those derived from the high-quality CPGs to ensure that recommendations were actionable in different contexts. Most of these provincial recommendations were consistent with those already incorporated, though there were some regional differences. For example, provincial guidance differed in regard to the criteria that should be used to determine the appropriate screening modality for an individual at elevated risk for colon cancer, all of which were reasonable based upon the high-quality guidance. Any jurisdictional nuances, such as differences in frequency of screening, were captured and indicated in the harmonized recommendations. Across all topics, individual and family risk factors (e.g., genetics, pre-existing medical conditions [personal or family], ethnic background) were incorporated to ensure that recommendations specific to high-risk and average-risk individuals were clear [11].

In Step 1, each topic-review team reviewed the international and national CPG recommendations identified through Phase 1 (see Fig. 1). As part of this first step, members were also asked to identify any high level international and national guidelines of which they were aware that were not included in their review. In Step 2, international and national clinical recommendations that reached consensus for inclusion were compared with provincial recommendations and policies to determine whether they were implementable in the 4 Canadian provinces of interest. For example, at the time of these discussions, the use of low-dose computed tomography (CT) scan for lung cancer screening, flexible sigmoidoscopy for average-risk colorectal cancer screening, and HPV testing for cervical cancer screening each varied among the different provinces. Any provincial clinical recommendations that differed from those already included and which were necessary to comply with a province’s approach to CCDPS were accepted for inclusion, noting the specific relevance to that province.

Finally, in Step 3, the topic-review teams reviewed the tools and resources previously included in the BETTER program [6, 11] alongside new tools/resources that were identified through our search strategy. Teams reflected on the tools’ clarity (i.e., use of unambiguous, understandable language), applicability (e.g., to diverse populations, patients 40–69 years of age), and clinical usefulness (e.g., for primary care settings and PPs). Validity was also considered for assessment tools (e.g., Patient Health Questionnaire 2-item (PHQ-2) [27] screen for depression, QRISK3 [28] cardiovascular disease risk calculator, and General Practice Physical Activity Questionnaire (GPPAQ) [29] screen for aerobic physical activity). Through this process, teams assembled updated, relevant, and useful clinician and/or patient-facing tools and resources to support the implementation of recommendations.

### Phase 3: refinement of the BETTER toolkit

The final CPG recommendations selected for inclusion were synthesized and harmonized using succinct, clear, and actionable language while considering PCPs’ and PPs’ scope of practice, role in patient care, and linkages to community resources. These recommendations were then used to refine the existing BETTER toolkit to assist PCPs and PPs with the implementation of recommendations. The toolkit provides PCPs and patients with the tools and resources needed to: 1) evaluate the individual patient’s risks for cancer and chronic disease, 2) identify CCDPS action(s) relevant to the patient, and 3) educate and prevent cancer and chronic diseases through a process of shared decision-making, resulting in a personalized ‘prevention prescription’ and actionable goals for the patient [11].

## Results

### Search results and clinical recommendations

**Fig. 2:**
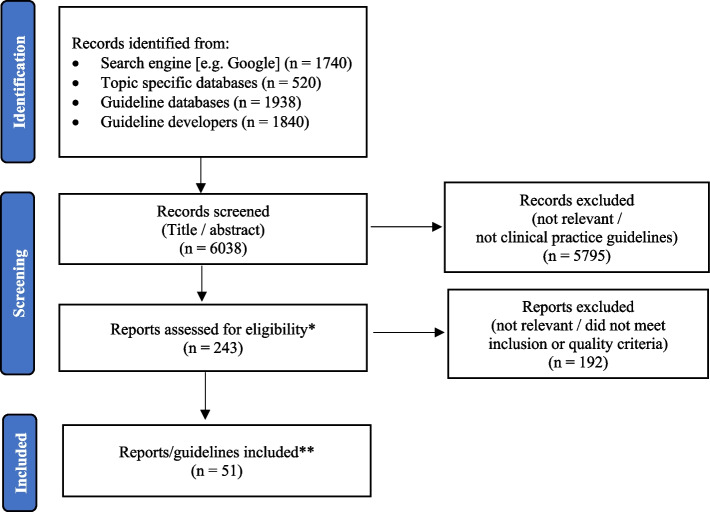
PRISMA diagram *Guidelines reviewed in full text. Initial quality appraisal completed using two key items assessing ‘Rigour of Development’ in the AGREE II instrument (items 7 and 12). A score of 5 or more in these domains typically indicates that the criteria is satisfied, however, guidelines with domain scores of least a 3 were considered for further appraisal** Appraised using full, 23-item, AGREE II Instrument. An additional 22 provincial guidelines were identified and included for review in Phase 2

The titles and abstracts of 6,038 CPGs identified through the search strategy were reviewed for relevance to CCDPS and primary care settings. Of these, 243 guidelines were retrieved, considered in full-text, and underwent initial quality appraisal. CPGs that passed the initial quality appraisal and that were further assessed using the full AGREE II received an overall quality score between 0 and 7, where a higher score indicates higher overall quality, with 7 indicating a CPG of the highest quality [26]. Thirteen percent of the CPGs assessed using the full AGREE II received an overall quality score of 4, 28% received a score of 5, 55% received a score of 6, and 4% received a score of 7 (see Appendix 3 for full AGREE II scores, including scores for overall quality). After excluding those that did not meet the inclusion or quality criteria, Phase 1 yielded 51 CPGs which provided current, relevant CCDPS recommendations for the general population (see Fig. 2). A summary of the search results is provided in Appendix 2.

Supplementary searches identified an additional 22 guidelines by provincial organizations in Alberta, Ontario, Nova Scotia, and Newfoundland and Labrador, which were also reviewed by the BETTER CWG in Phase 2. Discussions within topic-review teams and the larger CWG determined that 3 of the new topics proposed (COPD, hepatitis C, and drug use) did not have sufficient evidence for inclusion (e.g., lack of prevention and screening CPGs for the topic, focus on diagnosis, treatment, and/or management) and thus were excluded from the final recommendations, resulting in 16 topics included in the program – 3 new: osteoporosis/bone health, cannabis use, and vaping/e-cigarette use. Notably, there were no CPGs published between 2016 and 2021 that met our criteria for inclusion on the topic of depression. The decision made by the CWG was to include a recommendation to screen for depression since this was already a part of the BETTER program. The final recommendations and resources/tools included in the BETTER program are presented in Tables 2 and 3, respectively.

**Table 2:** Summary of primary prevention and screening recommendations for adults 40–69 years of age

Topic	Recommendation
Alcohol Screening Recommendations [31–38]	• All adults: Annual screen for alcohol consumption • Alcohol drinking recommendations are: o Limit alcohol o Adults: ≤ 6 drinks per week o No binge drinking (≥ 4 drinks for men and ≥ 3 drinks for women in one sitting)
Breast Cancer Screening Recommendations [39–45]	• Screen women without personal history of, or elevated risk for, breast cancer every 2 years with mammogram starting at age 50 • Annual MRI screening in addition to mammography starting at age 30 for women in very high-risk population subgroups as defined below (if meet criteria refer to PCP to verify risk and appropriate screening): o Known mutation in BRCA1, BRCA2 or other gene predisposing to a markedly elevated breast cancer risk OR o Untested first-degree relative of a carrier of such a gene mutation OR o FH consistent with a hereditary breast cancer syndrome and estimated personal lifetime cancer risk > 25%OR o Women who received chest radiation (not chest x-ray) before age 30 and at least 8 years previously (e.g. as treatment for Hodgkin’s Lymphoma) o AB – women meeting any of the criteria above should receive an annual MRI, mammogram, and clinical breast exam starting at age 30 • Consider referral to genetics if any of the following: o Personal and/or FH of ovarian cancer any age (epithelial) o Family member with BRCA1/BRCA2 mutation o High risk ethnicity (e.g. Ashkenazi Jewish, Icelandic) + personal and/or FH of hereditary breast and ovarian related cancers (breast, ovarian, male breast, pancreatic, prostate with Gleason Score ≥ 7) o ON – women meeting any of the criteria above should be refered to the Ontario Breast Screening Program • For women with one or two first-degree relatives with invasive breast cancer, but who do not meet the criteria for referral to genetics or MRI screening: o Annual mammography starting 5 to 10 years younger than the youngest case in the family, but no earlier than age 25 and no later than age 40 o Annual clinical breast examination starting at age 25
Cannabis Use Screening Recommendations [46]	• All adults: screen annually for non-prescribed cannabis use (including edibles and oils) • Encourage reduction/cessation • Refer for counseling, program, or discussion with PCP
Cardiovascular Disease Recommendations [9, 36, 47–49]	• All adults: blood pressure should be measured annually o Target for individuals diagnosed with DM or CKD = ≤ 130/80 o Target for individuals without a DM or CKD diagnosis = ≤ 140/90 o Refer individuals above these targets to PCP for diagnosis and management • Perform CVD risk assessment in persons with Type 2 Diabetes, men > 40 years of age and women > 50 years of age or post-menopausal (exclude individuals with familial hypercholesterolemia) o Recommend every 3–5 years if low/moderate risk (< 10%) or every year if high risk (≥ 10%) or risks change • Use QRISK3 calculator for CVD risk or choose a CVD risk calculator and use it consistently • Refer to PCP to discuss statin if: o moderate to high risk on QRISK3 or CVD calculator used • Refer to PCP to discuss ACE/ARB if: o Individual has personal history of DM or CAD or CVD or PVD or HTN or KD (eGFR < 60 or microalbuminuria) AND o Individual is not already on an ACE/ARB • Recommend statins for all adults > 40 with DM
Cervical Cancer Screening Recommendations [50–55]	• Women at average risk: routine screening for cervical cancer using a Pap test every 3 years or HPV test every 5 years • Women following a hysterectomy with removal of the cervix who have no history of a high-grade precancerous lesion should not be screened • Women following a hysterectomy with removal of the cervix with a history of a high-grade precancerous lesion should be screened annually using a vault smear • Women who are immunocompromised (e.g., on immune-suppressing medications, post-organ transplant, HIV positive, or undergoing cancer treatment) should receive annual screening using a pap test
Colorectal Cancer Screening Recommendations [56–63]	• Adults at average risk: screen for colorectal cancer with FIT (or FOBT) every 2 years or flexible sigmoidoscopy every 5 years or colonoscopy every 10 years starting at age 50 o ON: individuals who choose to be screened with flexible sigmoidoscopy should be screened every 10 years • Adults at increased (high) risk should be screened as follows: o Single first degree relative with CRC at age ≥ 60 ▪ AB – FIT starting at 40 ▪ NL – FIT starting at 50 ▪ NS – FIT or FOBT or colonoscopy starting at 40 ▪ ON – Colonoscopy every 5 years or as directed starting at age 50 or 10 years younger than the youngest case in the family o Single first degree relative with CRC at age < 60 or two first degree relatives with CRC at any age ▪ AB – Colonoscopy starting at 40 or 10 years younger than the youngest case in the family (repeat as indicated) ▪ NL & ON – Colonoscopy starting at 50 or 10 years younger than the youngest case in the family ▪ NS – Colonoscopy every 5 years starting at age 40 or 10 years younger than the youngest case in the family o Single second degree relative with CRC diagnosis at age < 50 – colonoscopy starting at 50 (repeat as directed by findings) o Personal history of Crohn’s, UC, FAP, HNPCC, LS – colonoscopy at discretion of GI o Carrier of mutation in LS gene or untested first degree relative of a LS mutation carrier – colonoscopy every 1–2 years starting at age 20–25 or 2–5 years younger than the youngest case in the family if that diagnosis was made at age < 25, whichever is earlier • Refer individuals with suspected LS to PCP to discuss genetics referral
Depression Screening Recommendations [64–67]	• Use PHQ-2 to screen annually for depression in all individuals
Diabetes Screening Recommendations [49, 68–73]	• Adults at average risk: screening for diabetes using FBS/FBG or HbA1c should be performed every 3 years starting at 40 years of age o If FBS/FBG is 6.0–6.9 mmol/L or HbA1c is 6.0–6.4%, repeat FBS/FBG or HbA1c at 6-months ▪ If no change, refer to PCP for discussion of physical activity, diet, and pharmacotherapy o If FBS is ≥ 7 mmol/L or HbA1c is ≥ 6.5% refer to PCP for management and discussion of physical activity, diet, and pharmacotherapy • Recommend annual screening for high-risk individuals who meet at least one of the following: o CKD o CVD o Family history of a first-degree relative with type 2 diabetes o From a community of color – African, Caribbean, Black, East Asian, Southeast Asian, South Asian, and Latin o History of gestational diabetes o HTN or elevated BP or on HTN medication o Hyperlipidemia o Low socio-economic status o Medications (glucocorticoids, atypical antipsychotics, HAART) o Of African, Arab, Asian, Hispanic, Indigenous, or South Asian descent o Obesity (BMI ≥ 30) or abdominal obesity (High waist circumference) o Past impaired FBS/FBG or HbA1c o Polycystic ovarian disease • Adults with a DM diagnosis o Recommend retina screening every 1–2 years o Recommend CKD screening using random urine ACR and serum creatinine converted into an eGFR every year
Lung Cancer Screening [74–76]	• Screen for lung cancer among adults ≥ 55 years of age with at least a 30 pack-year smoking history, who currently smoke or quit smoking less than 15 years ago, with low-dose CT scan every year up to three consecutive years o ON – adults ≥ 55 years of age with at least a 20-year smoking history should be referred to PCP for referral to the Ontario Lung Screening Program • *Screening should only be done in health care settings with access to screening resources, expertise in early diagnosis and treatment of lung cancer*
Nutrition/Diet Screening Recommendations [9, 33, 36, 47, 48, 68, 77, 78]	• All adults: screen for healthy eating behaviours • Encourage Mediterranean-style diet with variety of vegetables, fruit, healthy proteins and unsaturated fats • Encourage limiting intake of refined sugar • In individuals at risk for type 2 diabetes encourage dietary patterns used to reduce risk of diabetes: Mediterranean-style, DASH, AHEI
Obesity Screening Recommendations [79–82]	• For individuals who have indicated willingness to discuss weight and after asking permission, screen all individuals with height, weight, BMI o For those with BMI ≥ 30 refer to PCP for discussion of root cause assessment, lifestyle, risk stratification with Edmonton Obesity Staging Scale, optimization of medications, and need for referral • Measure WC if BMI 25.0—29.9 o For those with high WC refer to PCP for discussion of root cause assessment, lifestyle, risk stratification with Edmonton Obesity Staging Scale, optimization of medications, and need for referral o High waist circumference is defined as: ▪ For South Asian, Chinese, Malay, Asian Indian, Japanese, or South and Central Amerian ethnic backgrounds: • ≥ 90 cm for Men • ≥ 80 cm for Women ▪ For Europid, Sub-Saharan African, Eastern Mediterranean, and Middle East (Arab) ethnic backgrounds; • ≥ 94 cm for Men • ≥ 80 cm for Women ▪ For North American ethnic background: • ≥ 102 cm for Men • ≥ 88 cm for Women • These cutoffs may also be used for the Europid ethnic group ▪ Europid includes ethnic backgrounds from Europe, Northeast Atlantic, North Africa, the Horn of Africa, West Asia, and Central Asia
Osteoporosis/Bone Health Screening Recommendations [33, 77, 83–88]	• Women at average risk: screen for osteoporosis among women ≥ 65 years of age if no bone density screen has been completed since age 60 • Recommend bone mineral density screening in adults ≥ 50 years of age who present with a fragility fracture after age 40 and is at risk for future fractures • Recommend considering a fracture risk assessment for high-risk individuals ≥ 50 years of age who meet at least one of the following (particularly in the presence of other risk factors): o BMI < 20 kg/m^2^ o Consume > 3 drinks containing alcohol per day o Current smoker* o Living with HIV o Low bone mineral density on DXA/DEXA o On long-term antidepressant treatment (in particular, SSRIs) o Parental history of osteoporosis o Parental history of hip fracture* o Prolonged use of glucocorticoids (at least 3-months in the previous year at a prednisone-equivalent dose > 7.5 mg daily)* o Taking proton pump inhibitors o Using thiazolidinediones (antidiabetic medication) o Personal history of: ▪ a neurological disease (including Alzheimer’s disease, Parkinson’s disease, multiple sclerosis and stroke) or ▪ asthma or ▪ chronic liver disease or ▪ diabetes or ▪ epilepsy taking enzyme-inducing antiepilectic agents or ▪ inflammatory bowel disease* or malabsorption* or ▪ hyperparathyroidism* or other endocrine* disases or ▪ moderate to severe chronic kidney disease (eGFR < 60 ml/min/1.73 m2) or ▪ rheumatoid arthritis* or systemic lupus erythematosus* o Men ▪ Personal history of prostate cancer and taking GnRH agonists o Women ▪ History of previously untreated early menopause ▪ Taking aromatase inhibitors* o AB – adults ≥ 50 years of age who meet at least one of the * criteria above should be considered for bone mineral density screening
Physical Activity Recommendations [9, 33, 36, 47–49, 68, 77, 78, 89, 90]	• All adults: 150–300 min of moderate intensity physical activity or 75–150 min of vigorous intensity physical activity per week, with activity time divided up so that a person is active most days of the week o If a person is unable to perform moderate intensity physical activity, they should exercise up to their maximum safe capacity • All adults: strength or resistance training ≥ 2 days per week
Prostate Cancer Screening Recommendations [91–95]	• Do not recommend routine PSA screening • Provide individuals with information about prostate cancer screening and refer to PCP if the individual is: o ≥ 45 years old AND from a Black ethnic background or has a family history of a first degree relative with a prostate cancer diagnosis OR o ≥ 50 years old
Tobacco Use, Vaping/E-cigarette Screening Recommendations [9, 33, 36, 47, 49, 75, 96–99]	• All adults: screen annually for commercial tobacco use (cigarettes, tobacco products) and nicotine use (vaping/e-cigarettes; non-prescribed) o Does not include tobacco used for ceremonial purposes • Encourage reduction/cessation • Refer for counseling, program, or discussion with PCP

*AB* Alberta, *ACE* Angiotensin converting enzyme, *ACR* Albumin to creatinine ratio, *AHEI* Alternate Healthy Eating Index, *ARB* Angiotensin II receptor blocker, *BMI* Body mass index, *BRCA* Breast cancer gene, *CAD* Coronary artery disease, *CKD* Chronic kidney disease, *CRC* Colorectal cancer, *CVD* Cardiovascular disease, *CT* Computed tomography, *DASH* Dietary Approaches to Stop Hypertension, *DM* Diabetes mellitus, *DXA/DEXA* Dual energy x-ray absorptiometry, *eGFR* estimated glomerular filtration rate, *FAP* Familial adenomatous polyposis, *FBS* Fasting blood sugar, *FBG* Fasting blood glucose, *FH* Family history, *FIT* Fecal immunochemical test, *FOBT* F*ecal occult blood test*, *GI *Gastroenterologist*, GnRH* Gonadotropin-releasing hormone, *HAART* Highly active antiretroviral therapy, *HbA1c* Hemoglobin A1c, *HNPCC* Hereditary non-polyposis colorectal cancer, *HTN* Hypertension, *HPV* Human papillomavirus, *KD* Kidney disease, *LS* Lynch syndrome, *MRI* Magnetic resonance imaging, *NL* Newfoundland and Labrador, *NS* Nova Scotia, *ON* Ontario, *PCP* Primary care provider, *PHQ-2* Patient Health Questionnaire 2-item, *PSA *Prostate-specific antigen*, PVD* Peripheral vascular disease, *SSRI* Selective serotonin reuptake inhibitor, *UC* Ulcerative colitis, *WC* Waist circumference

**Table 3:** BETTER program resources and tools list

Topic Scope	Name	Tool Type	URL	Jurisdictional Availability
Alcohol	Alcohol or drug use tools and programs	Website	https://mha.nshealth.ca/en/topics/symptoms/alcohol-or-drug-use	Nova Scotia
Alcohol	Alcohol Screening, Brief Intervention & Referral: A Clinical Guide	PDF handout	https://www.rcdhu.com/wp-content/uploads/2017/03/Alcohol-Screening-Brief-Intervention-Referral.pdf	National
Alcohol	Alcohol Use Disorders Identification Test (AUDIT-C)	PDF handout	https://www.mirecc.va.gov/cih-visn2/Documents/Clinical/AUDIT-C.pdf	National
Alcohol	Breaking Free: Confidential wellness and recovery support program	Online program	https://www.breakingfreeonline.ca/	Ontario and Newfoundland & Labrador
Alcohol	Get Help with Substance Use	Online tool	https://www.canada.ca/en/health-canada/services/substance-use/get-help-problematic-substance-use.html	National
Alcohol	Limit alcohol	Website	https://cancer.ca/en/cancer-information/reduce-your-risk/limit-alcohol	National
Alcohol	Limit alcohol to reduce your cancer risk	PDF handout	https://prevent.cancer.ca/wp-content/uploads/2019/05/CMPR_1pgr_LimitAlcohol-EN.pdf	National
Alcohol	National Institute on Alcohol Abuse and Alcoholism website	Website	www.niaaa.nih.gov	National
Alcohol	Questions about cutting back on alcohol	PDF handout	https://www.rxfiles.ca/rxfiles/uploads/documents/alcohol-patient-booklet.pdf	National
Alcohol	Rethink your drinking	Website	http://www.rethinkyourdrinking.ca/	National
Alcohol	Single Item Alcohol Screening Questionnaire (SASQ)	PDF handout	https://www.icsi.org/wp-content/uploads/2021/11/Brief-Screen-FINAL.pdf	National
Bone health/ Osteoporosis	Fracture Risk Assessment (FRAX) Tool for Canada	Online tool	https://www.sheffield.ac.uk/FRAX/tool.aspx?country=19	National
Bone health/ Osteoporosis	Fragility Fracture Decision Aid	Online tool	https://frax.canadiantaskforce.ca/	National
Bone health/ Osteoporosis	How much calcium do we need?	Website	https://osteoporosis.ca/calcium-requirements/	National
Bone health/ Osteoporosis	Nutrition Guide for Calcium & Vitamin D for Prevention and Treatment of Osteoporosis	PDF handout	https://www.albertahealthservices.ca/assets/info/nutrition/if-nfs-ng-calcium-and-vitamin-d.pdf	Alberta
Bone health/ Osteoporosis	Qfracture	Online tool	https://qfracture.org/	National
Bone health/ Osteoporosis	Take Action- Prevent a Fall Before it Happens	PDF handout	https://myhealth.alberta.ca/Alberta/AlbertaDocuments/take-action-fall-prevention-booklet.pdf	Alberta
Breast Cancer	Breast Cancer Screening – it’s never this obvious	PDF handout	https://www.cancercareontario.ca/sites/ccocancercare/files/assets/OBSPBrochureNotObvious.pdf	Ontario
Breast Cancer	Breast Cancer Screening & Diagnosis Pathway Map	PDF handout	https://www.cancercareontario.ca/sites/ccocancercare/files/assets/BreastCancerScreeningandDiagnosisPathwayMap.pdf	Ontario
Breast Cancer	Breast Cancer Screening for Women at High Risk	Website	https://www.cancercareontario.ca/en/guidelines-advice/cancer-continuum/screening/breast-cancer-high-risk-women	Ontario
Breast Cancer	Breast Cancer Screening for Women not at Increased Risk, Age 40–49	PDF handout	**EN:** https://canadiantaskforce.ca/wp-content/uploads/2020/04/CTFPHC_Breast_Cancer_1000_Person-Single-Pages-40-49-Final.pdf **FR:** https://canadiantaskforce.ca/wp-content/uploads/2020/04/CTFPHC_Breast_Cancer_1000_Person-Single-Pages-French_40-49_Final.pdf	National
Breast Cancer	Breast Cancer Screening for Women not at Increased Risk, Age 50–59	PDF handout	**EN:** https://canadiantaskforce.ca/wp-content/uploads/2020/04/CTFPHC_Breast_Cancer_1000_Person-Single-Pages-50-59-Final.pdf **FR:** https://canadiantaskforce.ca/wp-content/uploads/2020/04/CTFPHC_Breast_Cancer_1000_Person-Single-Pages-French_50-59.pdf	National
Breast Cancer	Breast Cancer Screening for Women not at Increased Risk, Age 60–69	PDF handout	**EN:** https://canadiantaskforce.ca/wp-content/uploads/2020/04/CTFPHC_Breast_Cancer_1000_Person-Single-Pages-60-69-Final.pdf **FR:** https://canadiantaskforce.ca/wp-content/uploads/2020/04/CTFPHC_Breast_Cancer_1000_Person-Single-Pages-French_60-69.pdf	National
Breast Cancer	Breast Cancer Screening for Women not at Increased Risk, All Ages	PDF handout	EN: https://canadiantaskforce.ca/wp-content/uploads/2020/04/CTFPHC_Breast_Cancer_1000_Person-Final.pdfFR: https://canadiantaskforce.ca/wp-content/uploads/2020/04/CTFPHC_Breast_Cancer_1000_Person_All_Ages_French_Final.pdf	National
Breast Cancer	Breast Screening	PDF handout	https://www.cancercareontario.ca/sites/ccocancercare/files/assets/CancerScreeningToolkitBreastScreening.pdf	Ontario
Breast Cancer	Breast screening—do I really need a mammogram?	PDF handout	https://screeningforlife.ca/wp-content/uploads/2019/12/BREAST001-Brochure-Do-I-Really-Need-Rev-2021-07.pdf?pdf=BREAST001-Brochure-Do-I-Really-Need-Rev-2021-07	Alberta
Breast Cancer	Breast screening—the basics of breast cancer	PDF handout	**EN:** https://screeningforlife.ca/wp-content/uploads/2019/12/BREAST003-Brochure-The-Basics-Rev-2021-07.pdf?pdf=BREAST003-Brochure-The-Basics-Rev-2021-07 **FR:** https://screeningforlife.ca/wp-content/uploads/2019/12/ABCSP-The-Basics-of-Breast-Cancer-French-Aug-2019.pdf?pdf=ABCSP+The-Basics-of-Breast-Cancer-French-Aug-2019	Alberta
Breast Cancer	Breast Screening Program	Website	https://cancercare.easternhealth.ca/prevention-and-screening/breast-screening-program/	Newfoundland & Labrador
Breast Cancer	Get Screened (for breast cancer)	Website	https://screeningforlife.ca/breast/get-screened/	Alberta
Breast Cancer	Get screened for breast cancer	Website	https://cancer.ca/en/cancer-information/find-cancer-early/get-screened-for-breast-cancer	National
Breast Cancer	Guideline Recommendation: Breast Cancer Screening for Women Not at Increased Risk, Age 40–49	PDF handout	**EN:** https://canadiantaskforce.ca/wp-content/uploads/2020/06/CTFPHC_Breast_Cancer_Shared_Decision_Making_Tool_40-49_Final.pdfFR**:** https://canadiantaskforce.ca/wp-content/uploads/2020/08/CTFPHC_Breast_Cancer_Shared_Decision_Making_Tool_French_40-49_FR.pdf	National
Breast Cancer	Guideline Recommendation: Breast Cancer Screening for Women Not at Increased Risk, Age 50–59	PDF handout	**EN:** https://canadiantaskforce.ca/wp-content/uploads/2020/06/CTFPHC_Breast_Cancer_Shared_Decision_Making_Tool_50-59_Final.pdf **FR:** https://canadiantaskforce.ca/wp-content/uploads/2020/08/CTFPHC_Breast_Cancer_Shared_Decision_Making_Tool_French_50-59.pdf	National
Breast Cancer	Guideline Recommendation: Breast Cancer Screening for Women Not at Increased Risk, Age 60–69	PDF handout	**EN:** https://canadiantaskforce.ca/wp-content/uploads/2020/06/CTFPHC_Breast_Cancer_Shared_Decision_Making_Tool_60-69_Final.pdf **FR:** https://canadiantaskforce.ca/wp-content/uploads/2020/08/CTFPHC_Breast_Cancer_Shared_Decision_Making_Tool_French_60-69.pdf	National
Breast Cancer	Hereditary Breast and Ovarian Cancer	PDF handout	https://geneticseducation.ca/wp-content/uploads/2013/12/GECKO-on-the-run-HBOC-FINAL-April-2016.pdf	National
Breast Cancer	Honouring the First Nations path of well-being: Breast Cancer Screening	PDF handout	**First Nations:** https://www.cancercareontario.ca/sites/ccocancercare/files/assets/ACCUBreastFactSheet-FN.pdf	Ontario
Breast Cancer	Honouring the Inuit path of well-being: Breast Cancer Screening	PDF handout	**Inuit:** https://www.cancercareontario.ca/sites/ccocancercare/files/assets/ACCUBreastFactSheet-Inuit_0.pdf	Ontario
Breast Cancer	Honouring the Métis path of well-being: Breast Cancer Screening	PDF handout	**Metis:** https://www.cancercareontario.ca/sites/ccocancercare/files/assets/ACCUBreastFactSheet-Metis.pdf	Ontario
Breast Cancer	IBIS Breast Cancer Risk Assessment Tool	Online tool	https://ibis.ikonopedia.com/	National
Breast Cancer	Know your breasts	Online tool	https://cancer.ca/en/cancer-information/find-cancer-early/know-your-body/know-your-breasts	National
Breast Cancer	Making An Informed Decision About Breast Cancer Screening for Women 50 and Older	PDF handout	https://screeningforlife.ca/wp-content/uploads/Informed-Decision-Making-Rev-2021-07.pdf?pdf=Informed-Decision-Making-Rev-2021-07	Alberta
Breast Cancer	My family health portrait	Online tool	http://kahuna.clayton.edu/jqu/FHH/html/index.html	National
Breast Cancer	Nova Scotia Breast Screening Program	Website	https://breastscreening.nshealth.ca/	Nova Scotia
Breast Cancer	Ontario Breast Screening Program (OBSP)	Program	https://www.cancercareontario.ca/en/cancer-care-ontario/programs/screening-programs/ontario-breast-obsp	Ontario
Breast Cancer	Ontario Breast Screening Program (OBSP) Guidelines Summary	PDF handout	https://www.cancercare.on.ca/common/pages/UserFile.aspx?fileId=349883	Ontario
Breast Cancer	Point of Care Hereditary Breast and Ovarian Cancer (GEC-KO)	PDF handout	https://geneticseducation.ca/uploads/HBOC%20triage%20tool%20Part%201%20and%202%20-%20Final%20-July2019.pdf	National
Breast Cancer	Red Flags Point of Care tool (GEC-KO)	PDF handout	http://geneticseducation.ca/wp-content/uploads/2013/03/Red-flags-POC-quick-reference.pdf	National
Breast Cancer	Risks for breast cancer	Website	https://cancer.ca/en/cancer-information/cancer-types/breast/risks	National
Breast Cancer	Should I be screened for breast cancer?	PDF handout	**EN:** https://cdn.cancer.ca/-/media/files/cancer-information/resources/publications/should-i-be-screened-for-breast-cancer/ccs-breastcancerscreening-en-hires.pdf **FR:** https://cdn.cancer.ca/-/media/files/cancer-information/resources/publications/should-i-be-screened-for-breast-cancer/ccs-breastcancerscreening-fr-hires.pdf	National
Breast Cancer	Symptoms of breast cancer	Online tool	https://cancer.ca/en/cancer-information/cancer-types/breast/signs-and-symptoms	National
Cancer—General	Canadian Cancer Society website	Website	https://cancer.ca/en/	National
Cancer—General	Cancer Care for Health Care Professionals	Website	https://cancercare.easternhealth.ca/health-care-professionals/	Newfoundland & Labrador
Cancer—General	Cancer Care Ontario webpage	Website	https://www.cancercareontario.ca/en	Ontario
Cancer—General	Cancer Care Program: Tips for Protecting Your Health	Website	https://library.nshealth.ca/Cancer/Prevention	Nova Scotia
Cancer—General	Cancer Prevention and Screening	Website	https://cancercare.easternhealth.ca/prevention-and-screening/	Newfoundland & Labrador
Cancer—General	Cancer Screening in Alberta	PDF handout	https://screeningforlife.ca/wp-content/uploads/AHS_CancerScreeninginAB_Revised.pdf?pdf=AHS_CancerScreeninginAB_Revised	Alberta
Cancer—General	Cancer Screening Programs	Website	https://library.nshealth.ca/Cancer/Screening	Nova Scotia
Cancer—General	Check your family history	Website	https://cancer.ca/en/cancer-information/reduce-your-risk/check-your-family-history	National
Cancer—General	Eat well	Website	https://cancer.ca/en/cancer-information/reduce-your-risk/eat-well	National
Cancer—General	Explore prevention program	Website	https://cancer.ca/en/cancer-information/reduce-your-risk/explore-prevention-programs	National
Cancer—General	First Nations, Inuit, Métis and Urban Indigenous Cancer Screening Resources	Website	https://www.cancercareontario.ca/en/get-checked-cancer/indigenous-cancer-screening-resources	Ontario
Cancer—General	Get Checked for Cancer	Website	https://www.cancercareontario.ca/en/get-checked-cancer	Ontario
Cancer—General	Have a healthy body weight	Website	https://cancer.ca/en/cancer-information/reduce-your-risk/have-a-healthy-body-weight	National
Cancer—General	Healthier Together website	Website	https://www.healthiertogether.ca/health-conditions/cancer/	Alberta
Cancer—General	It's My Life! Stop Cancer Before it Starts	Website	EN: https://itsmylife.cancer.ca/ FR: https://cestmavie.cancer.ca/	National
Cancer—General	Limit alcohol	Website	https://cancer.ca/en/cancer-information/reduce-your-risk/limit-alcohol	National
Cancer—General	Live smoke-free	Website	https://cancer.ca/en/cancer-information/reduce-your-risk/live-smoke-free	National
Cancer—General	My CancerIQ: Learn Your Risk	Online tool	https://www.mycanceriq.ca/	Ontario
Cancer—General	My family health portrait	Online tool	http://kahuna.clayton.edu/jqu/FHH/html/index.html	National
Cancer—General	MyHealthAlberta	Website	https://myhealth.alberta.ca/health/healthy-living/Pages/default.aspx	Alberta
Cancer—General	Personalized Cancer Genomic Medicine Resource Toolkit	PDF handout	https://geneticseducation.ca/uploads/cancer_toolkit_gecko_june_2018.pdf	National
Cancer—General	Red Flags Point of Care tool (GEC-KO)	PDF handout	http://geneticseducation.ca/wp-content/uploads/2013/03/Red-flags-POC-quick-reference.pdf	National
Cancer—General	Red Flags to identify patients with risk of a hereditary cancer syndrome	PDF handout	https://geneticseducation.ca/wp-content/uploads/2014/02/POC-Hereditary-Cancer-Syndrome-triage-tool-FINAL.pdf	National
Cancer—General	Reduce your risk	Website	https://cancer.ca/en/cancer-information/reduce-your-risk	National
Cancer—General	Screening for Life website	Website	http://screeningforlife.ca/	Alberta
Cancer—General	Screening in 2SLGBTQI + communities	Website	https://cancer.ca/en/cancer-information/find-cancer-early/screening-in-lgbtq-communities	National
Cancer—General	What can I do to reduce my risk of cancer?	Website	https://www.canada.ca/en/public-health/services/chronic-diseases/cancer/what-reduce-risk-cancer.html	National
Cancer—General	What is Cancer? What is Screening?	PDF handout	https://www.cancercareontario.ca/sites/ccocancercare/files/assets/CancerScreeningToolkitWhatIsCancerScreening.pdf	Ontario
Cannabis	10 ways to reduce risks to your health when using cannabis	PDF handout	https://www.camh.ca/-/media/files/pdfs---reports-and-books---research/canadas-lower-risk-guidelines-cannabis-pdf.pdf	National
Cannabis	7 Things You Need to Know about Edible Cannabis	PDF handout	https://www.ccsa.ca/sites/default/files/2019-06/CCSA-7-Things-About-Edible-Cannabis-2019-en.pdf	National
Cannabis	A Guide to Cannabis for Older Adults	PDF handout	https://www.ccsa.ca/sites/default/files/2020-07/CCSA-Cannabis-Use-Older-Adults-Guide-2020-en.pdf	National
Cannabis	Be in the know about legal cannabis in Nova Scotia	Website	https://novascotia.ca/cannabis/	Nova Scotia
Cannabis	Cannabinoids: Overview	PDF handout	https://www.rxfiles.ca/RxFiles/uploads/documents/Pain-QandA-cannabinoids.pdf	National
Cannabis	Cannabis and Your Health: 10 ways to reduce risk with using	PDF handout	https://www.camh.ca/-/media/files/pdfs---reports-and-books---research/canadas-lower-risk-guidelines-cannabis-poster.pdf	National
Cannabis	Cannabis, heart disease and stroke	Website	https://www.heartandstroke.ca/heart-disease/risk-and-prevention/lifestyle-risk-factors/heavy-alcohol-use/cannabis-heart-disease-and-stroke	National
Cannabis	Knowing Your Limits with Cannabis: A Practical Guide to Assessing Your Cannabis Use	Website	https://www.ccsa.ca/sites/default/files/2022-04/CCSA-Knowing-Your-Limits-with-Cannabis-Guide-2022-en.pdf	National
Cannabis	Questions about cannabis and the answers that may surprise you: A booklet for people thinking about starting medical cannabis	PDF handout	https://www.rxfiles.ca/rxfiles/uploads/documents/Cannabis-Medical-Patient-Booklet.pdf	National
Cannabis	What you need to know if you choose to consume cannabis	PDF handout	https://www.canada.ca/content/dam/hc-sc/documents/services/drugs-medication/cannabis/resources/what-you-need-to-know-if-you-choose-to-consume-cannabis-eng.pdf	National
Cannabis	Your Cannabis Questions Answered	Website	https://www.gov.nl.ca/cannabis/	Newfoundland & Labrador
Cardiovascular Disease	Are you at risk for heart disease or stroke?	PDF handout	https://heartstrokeprod.azureedge.net/-/media/1-stroke-best-practices/resources/patient-resources/hs-f20-areyouatrisk-booklet-en-v3.ashx?rev=dfd751bf37b446bca8b1678591ec94a3	National
Cardiovascular Disease	DASH diet recipes	Website	http://www.mayoclinic.org/healthy-lifestyle/recipes/dash-diet-recipes/rcs-20077146	National
Cardiovascular Disease	Framingham Risk Score (FRS)	PDF handout	https://ccs.ca/app/uploads/2020/12/FRS_eng_2017_fnl1.pdf	National
Cardiovascular Disease	Healthy eating for healthy blood pressure	PDF handout	https://www.mountsinai.on.ca/care/fammed/patient-resources/hypertension/hypertension-nutrition.pdf	National
Cardiovascular Disease	How to Manage Your Cholesterol	PDF handout	https://heartstrokeprod.azureedge.net/-/media/pdf-files/iavc/health-information-catalogue/amgencholesterol_broch_en_web.ashx?la=en&rev=c8e68e5957c54322a85828229039ca00	National
Cardiovascular Disease	Hypertension Canada	Website	https://hypertension.ca	National
Cardiovascular Disease	In brief: Your guide to lowering your blood pressure with DASH	PDF handout	https://www.nhlbi.nih.gov/files/docs/public/heart/dash_brief.pdf	National
Cardiovascular Disease	Managing Your Blood Pressure	PDF handout	https://heartstrokeprod.azureedge.net/-/media/pdf-files/canada/health-information-catalogue/en-managing-your-blood-pressure.ashx?la=en&rev=44da5a0d78c040789e53b7a52864e849	National
Cardiovascular Disease	MyHealthAlberta		https://myhealth.alberta.ca/health/healthy-living/Pages/default.aspx	Alberta
Cardiovascular Disease	PEER Simplified Guideline: Prevention and Management of Cardiovascular Disease Risk in Primary Care	PDF handout	https://actt.albertadoctors.org/CPGs/Lists/CPGDocumentList/CVD-Risk-CPG.pdf	Alberta
Cardiovascular Disease	Prevention and Management of CVD Risk in Primary Care—Lipid Algorithm	PDF handout	https://actt.albertadoctors.org/CPGs/Lists/CPGDocumentList/CVD-Risk-Summary.pdf	Alberta
Cardiovascular Disease	QRisk3	Online tool	https://qrisk.org/three/	National
Cardiovascular Disease	Reducing Your Risk for Heart Attacks & Strokes	PDF handout	https://actt.albertadoctors.org/CPGs/Lists/CPGDocumentList/Reducing-CVD-Risk-Patient-Handout.pdf#search=reducing%20cvd%20risk%20patient	National
Cardiovascular Disease	Salt and Sodium: Get the Facts	PDF handout	https://www.mountsinai.on.ca/care/fammed/patient-resources/nutrition/salt-and-sodium-get-the-facts.pdf/at_download/file	National
Cardiovascular Disease	Statin Choice Decision Aid	Online tool	https://statindecisionaid.mayoclinic.org/	National
Cardiovascular Disease	The Absolute CVD Risk/ Benefit Calculator	Online tool	https://cvdcalculator.com/	National
Cervical Cancer	Cervical screening—do I really need a Pap test?	PDF handout	**EN:** https://screeningforlife.ca/wp-content/uploads/2019/12/ACCSP-Do-I-Really-Need-a-Pap-Test-Brouchure-Aug-20-2019.pdf?pdf=ACCSP+Do+I+Really+Need+a+Pap+Test+Brouchure+Aug+20+2019 **FR:** https://screeningforlife.ca/wp-content/uploads/2019/12/ACCSP-Do-I-Really-Need-a-Pap-Test-Brochure-French-Aug-2019.pdf?pdf=ACCSP+Do+I+Really+Need+a+Pap+Test+Brochure+French+Aug+2019	Alberta
Cervical Cancer	Cervical Screening Information	PDF handout	https://www.cancercareontario.ca/sites/ccocancercare/files/assets/CancerScreeningToolkitCervicalScreening.pdf	Ontario
Cervical Cancer	Cervical Screening Program	Website	https://cancercare.easternhealth.ca/prevention-and-screening/cervical-screening-program/	Newfoundland & Labrador
Cervical Cancer	Frequently Asked Questions about Cervical Cancer Screening—For Clinicians	PDF handout	**EN:** http://canadiantaskforce.ca/wp-content/uploads/2016/06/2013-cervical-cancer-clinician-faq-en.pdf **FR:** https://canadiantaskforce.ca/wp-content/uploads/2016/09/2013-cervical-cancer-clinician-faq-fr.pdf	National
Cervical Cancer	Frequently Asked Questions about Cervical Cancer Screening—For Patients	PDF handout	**EN:** http://canadiantaskforce.ca/wp-content/uploads/2016/05/2013-cervical-cancer-patient-faq-en.pdf **FR:** https://canadiantaskforce.ca/wp-content/uploads/2016/09/2013-cervical-cancer-patient-faq-fr.pdf	National
Cervical Cancer	Get screened (cervical cancer)	Website	https://screeningforlife.ca/cervical/get-screened/	Alberta
Cervical Cancer	Get screened for cervical cancer	Website	https://cancer.ca/en/cancer-information/find-cancer-early/get-screened-for-cervical-cancer	National
Cervical Cancer	Honouring the First Nations path of well-being: Cervical Cancer Screening	PDF handout	**First Nations:** https://www.cancercareontario.ca/sites/ccocancercare/files/assets/ACCUCervicalFactSheet-FN.pdf	Ontario
Cervical Cancer	Honouring the Inuit path of well-being: Cervical Cancer Screening	PDF handout	**Inuit:** https://www.cancercareontario.ca/sites/ccocancercare/files/assets/ACCUCervicalFactSheet-Inuit.pdf	Ontario
Cervical Cancer	Honouring the Métis path of well-being: Cervical Cancer Screening	PDF handout	**Metis:** https://www.cancercareontario.ca/sites/ccocancercare/files/assets/ACCUCervicalFactSheet-Metis.pdf	Ontario
Cervical Cancer	Ontario Cervical Screening Program (OCSP) Screening Recommendations Summary	PDF handout	https://www.cancercareontario.ca/en/system/files_force/derivative/OCSPScreeningGuidelines.pdf	Ontario
Cervical Cancer	Pap test brochure—Take a Closer Look	PDF handout	https://www.cancercareontario.ca/sites/ccocancercare/files/assets/OCSPRightTimeBrochure.pdf	Ontario
Cervical Cancer	Risk factors for cervical cancer	Website	https://cancer.ca/en/cancer-information/cancer-types/cervical/risks	National
Cervical Cancer	Should you be screened for cervical cancer?	PDF handout	http://canadiantaskforce.ca/wp-content/uploads/2016/05/2013-cervical-cancer-patient-algorithm-en.pdf	National
Cervical Cancer	Who should be screened for cervical cancer?	PDF handout	**EN:** http://canadiantaskforce.ca/wp-content/uploads/2016/06/2013-cervical-cancer-clinician-algorithm-en.pdf **FR:** https://canadiantaskforce.ca/wp-content/uploads/2016/09/2013-cervical-cancer-clinician-algorithm-fr.pdf	National
Colorectal Cancer	Colon Cancer Check (CCC)- Guide to Average Risk Screening with the Fecal Immunochemical Test (FIT) in Ontario	PDF handout	https://www.cancercareontario.ca/sites/ccocancercare/files/assets/H-FIT_PCC_2742_ClinicalToolForProviders.pdf	Ontario
Colorectal Cancer	Colon Cancer Information	PDF handout	https://www.cancercareontario.ca/sites/ccocancercare/files/assets/CancerScreeningToolkitColonScreening.pdf	Ontario
Colorectal Cancer	Colon Cancer Screening Program	Website	https://cancercare.easternhealth.ca/prevention-and-screening/colon-cancer-screening/	Newfoundland & Labrador
Colorectal Cancer	Colorectal Cancer Screening—Common Questions	PDF handout	https://screeningforlife.ca/wp-content/uploads/2019/12/ACRCSP-Brochure-Colorectal-Cancer-Screening-Common-Questions-Aug-2019.pdf?pdf=ACRCSP+Brochure+Colorectal+Cancer+Screening+Common+Questions+Aug+2019	Alberta
Colorectal Cancer	Does My Patient Need A Fecal Immunochemical Test (FIT)?	PDF handout	https://screeningforlife.ca/wp-content/uploads/2020/02/ACRCSP-Does-my-patient-need-a-FIT.pdf?pdf=ACRCSP+Does+my+patient+need+a+FIT	Alberta
Colorectal Cancer	Everything You Need to Know About Colon Cancer Screening	PDF handout	https://library.nshealth.ca/ld.php?content_id=35488441	Nova Scotia
Colorectal Cancer	Get screened (colorectal cancer)	Website	https://screeningforlife.ca/colorectal/get-screened/	Alberta
Colorectal Cancer	Get screened for colorectal cancer	Website	https://cancer.ca/en/cancer-information/find-cancer-early/get-screened-for-colorectal-cancer	National
Colorectal Cancer	Honouring the First Nations path of well-being: Colon Cancer Screening	PDF handout	**First Nations:** https://www.cancercareontario.ca/sites/ccocancercare/files/assets/ACCUColonFactSheet-FN.pdf	Ontario
Colorectal Cancer	Honouring the Inuit path of well-being: Colon Cancer Screening	PDF handout	**Inuit:** https://www.cancercareontario.ca/sites/ccocancercare/files/assets/ACCUColonFactSheet-Inuit.pdf	Ontario
Colorectal Cancer	Honouring the Métis path of well-being: Colon Cancer Screening	PDF handout	**Métis:** https://www.cancercareontario.ca/sites/ccocancercare/files/assets/ACCUColonFactSheet-Metis.pdf	Ontario
Colorectal Cancer	Lynch Syndrome point of care tool (GEC-KO)	Document	http://geneticseducation.ca/wp-content/uploads/2014/02/POC-LS-tool-Part1-and-2-FINAL-Oct-2014.pdf	National
Colorectal Cancer	MyHealthAlberta	Website	https://myhealth.alberta.ca/health/healthy-living/Pages/default.aspx	Alberta
Colorectal Cancer	Risk factors for colorectal cancer	Website	https://cancer.ca/en/cancer-information/cancer-types/colorectal/risks	National
Colorectal Cancer	Screening for Colorectal Cancer—Clinician Recommendation Table	PDF handout	**EN:** https://canadiantaskforce.ca/wp-content/uploads/2016/05/ctfphccolorectal-cancerrecommendation-tablefinal160121-1.pdf **FR:** https://canadiantaskforce.ca/wp-content/uploads/2016/09/ctfphccolorectal-cancerrecommendation-tablefrenchfinal.pdf	National
Colorectal Cancer	Screening for Colorectal Cancer—Patient Frequently Asked Questions	PDF handout	http://canadiantaskforce.ca/wp-content/uploads/2016/05/ctfphccolorectal-cancerpatient-faqfinal-updated160222.pdf	National
Colorectal Cancer	When to use the Fecal Immunochemical Test (FIT)	PDF handout	https://screeningforlife.ca/wp-content/uploads/2019/12/ACRCSP-When-to-use-FIT-flow-chart-Nov-2016.pdf?pdf=ACRCSP+When+to+use+FIT+flow+chart+Nov+2016	Alberta
Diabetes	Are you at risk?	PDF handout	https://guidelines.diabetes.ca/docs/patient-resources/are-you-at-risk.pdf	National
Diabetes	Canadian Diabetes Risk Questionnaire (CANRISK)	PDF handout	http://canadiantaskforce.ca/wp-content/uploads/2016/05/2012-type-2-diabetes-canrisk-en.pdf	National
Diabetes	Diabetes Fact Sheet	PDF handout	https://www.diabetes.ca/diabetescanadawebsite/media/managing-my-diabetes/tools%20and%20resources/diabetes-fact-sheet.pdf?ext=.pdf	National
Diabetes	Just the Basics—Tips for Healthy Eating	PDF handout	https://www.diabetes.ca/DiabetesCanadaWebsite/media/Managing-My-Diabetes/Tools%20and%20Resources/just-the-basics.pdf?ext=.pdf	National
Diabetes	Just the Basics—Tips for Healthy Eating (Aboriginal English version)	PDF handout	https://www.diabetes.ca/DiabetesCanadaWebsite/media/Managing-My-Diabetes/Tools%20and%20Resources/just-the-basics-aboriginal-english.pdf?ext=.pdf	National
Diabetes	Patient Finnish Diabetes Risk Score (FINDRISK)	PDF handout	https://canadiantaskforce.ca/wp-content/uploads/2016/05/2012-type-2-diabetes-patient-findrisc-en.pdf	National
Diabetes	Prediabetes Fact Sheet	PDF handout	https://www.diabetes.ca/diabetescanadawebsite/media/managing-my-diabetes/tools%20and%20resources/prediabetes-fact-sheet.pdf?ext=.pdf	National
Diabetes	Questions about Type 2 Diabetes and the answers that may surprise you	PDF handout	https://www.rxfiles.ca/rxfiles/uploads/documents/diabetes-patient-booklet.pdf	National
Diabetes	Screening for Type 2 Diabetes in the Adult Population 2012—FAQ for Patients	PDF handout	http://canadiantaskforce.ca/wp-content/uploads/2016/05/2012-type-2-diabetes-patient-faq-en.pdf	National
Diabetes	Type 2 Diabetes—The Basics	PDF handout	https://www.diabetes.ca/diabetescanadawebsite/media/managing-my-diabetes/tools%20and%20resources/type-2-diabetes-the-basics.pdf?ext=.pdf	National
Diabetes	Type 2 Diabetes (GECKO On-the-run)	PDF handout	https://geneticseducation.ca/wp-content/uploads/2014/07/GECKO-On-the-Run-Type-II-Diabetes-FINAL-Feb-April-2014.pdf	National
Hepatitis C	Recommendations on Hepatitis C Screening for Adults—Clinician FAQs	PDF handout	https://canadiantaskforce.ca/wp-content/uploads/2017/04/CTFPHC_Hepatitis-C_Clinician-FAQ_v10_FINAL-1.pdf	National
Lung Cancer	Learning about Lung Cancer Screening	Website	https://myhealth.alberta.ca/health/AfterCareInformation/pages/conditions.aspx?HwId=abq1915	Alberta
Lung Cancer	Lung Cancer Screening—Clinician FAQ	PDF handout	**EN:** http://canadiantaskforce.ca/wp-content/uploads/2016/05/ctfphclung-cancerclinician-faqfinalv2-1.pdf **FR:** https://canadiantaskforce.ca/wp-content/uploads/2016/09/ctfphclung-cancerclinician-faqfrenchv2.pdf	National
Lung Cancer	Lung Cancer Screening—Patient Tool—Benefits vs Harms	PDF handout	**EN:** http://canadiantaskforce.ca/wp-content/uploads/2016/05/ctfphclung-cancerharms-and-benefitsfinal.pdf **FR:** https://canadiantaskforce.ca/wp-content/uploads/2016/09/ctfphclung-cancerharms-and-benefitsfrench-1.pdf	National
Lung Cancer	Risks for lung cancer	Website	https://cancer.ca/en/cancer-information/cancer-types/lung/risks	National
Lung Cancer	Finding lung cancer early	Website	https://cancer.ca/en/cancer-information/cancer-types/lung/finding-cancer-early	National
Mental Health	Addiction & Mental Health Information for Health Professionals	Website	https://www.albertahealthservices.ca/info/Page11536.aspx	Alberta
Mental Health	BounceBack	Program	https://bounceback.cmha.ca/	National
Mental Health	Bridge the gapp	Program	https://www.bridgethegapp.ca/	National
Mental Health	Connex Ontario	Program	https://www.connexontario.ca/en-ca/	Ontario
Mental Health	Depression Information Sheet	PDF handout	https://www.nshealth.ca/sites/nshealth.ca/files/patientinformation/0766.pdf	Nova Scotia
Mental Health	Distress and Crisis Ontario	Online tool	https://www.dcontario.org/locations/	Ontario
Mental Health	Distress Thermometer—National Cancer Comprehensive Network (NCCN)	PDF handout	https://www.nccn.org/docs/default-source/patient-resources/nccn_distress_thermometer.pdf?sfvrsn=ef1df1a2_6	National
Mental Health	eMentalHealth—Mental Health Services, Help and Support in Your Community	Website	https://www.ementalhealth.ca/	National
Mental Health	Find mental health and addiction services in your community	Online tool	https://www.ontario.ca/page/mental-health-services	Ontario
Mental Health	Generalized Anxiety Disorder 7-item (GAD-7) scale	PDF handout	https://www.thenationalcouncil.org/wp-content/uploads/2021/04/GAD708.19.08Cartwright.pdf?daf=375ateTbd56	National
Mental Health	Health Line	Website	https://www.811healthline.ca/medical-advice-and-health-information/	Newfoundland & Labrador
Mental Health	Mental Health and Addictions Helplines and Resources	Website	https://www.gov.nl.ca/hcs/mentalhealth-committee/mentalhealth/	Newfoundland & Labrador
Mental Health	Mental Health Helpline	Website	https://www.albertahealthservices.ca/findhealth/Service.aspx?id=6810&serviceAtFacilityID=1047134	Alberta
Mental Health	Patient Health Questionnaire (PHQ-9)	PDF handout	https://agencymeddirectors.wa.gov/Files/AssessmentTools/13-PHQ-9%20form.pdf	National
Mental Health	Provincial Mental Health and Addictions Crisis Line	Website	https://mha.nshealth.ca/en	Nova Scotia
Mental Health	Recovery Colleges	Website	https://cmha.ca/what-we-do/national-programs/recovery-colleges/	National
Mental Health	Screening for Depression in Primary Care	PDF handout	https://canadiantaskforce.ca/wp-content/uploads/2016/05/2013-depression-clinician-algorithm-and-faq-en.pdf	National
Mental Health	Wellness Together Canada	Program	https://www.wellnesstogether.ca/en-CA	National
Nutrition/Diet	Affordable Healthy Eating—Eat well and save money	PDF handout	https://www.gov.nl.ca/healthyeating/affordable/wp-content/uploads/sites/3/2021/05/Affordable-Healthy-Eating-Final-Oct-2020.pdf	Newfoundland & Labrador
Nutrition/Diet	Canada's Food Guide: Eat Well. Live Well (Snapshot)	PDF handout	https://food-guide.canada.ca/artifacts/CFG-snapshot-EN.pdf	National
Nutrition/Diet	Canada's Food Guide: Eat Well. Live Well (Snapshot)—All Languages	PDF handout	https://www.canada.ca/en/health-canada/services/canada-food-guide/resources/snapshot/languages.html	National
Nutrition/Diet	Canada's Food Guide: Healthy eating recommendations	PDF handout	https://food-guide.canada.ca/sites/default/files/artifact-pdf/HEPs-Guide-nw-en.pdf	National
Nutrition/Diet	Eat well	Website	https://cancer.ca/en/cancer-information/reduce-your-risk/eat-well	National
Nutrition/Diet	Eating well for weight and health	PDF handout	https://www.albertahealthservices.ca/assets/info/nutrition/if-nfs-eating-well-for-weight-and-health.pdf	National
Nutrition/Diet	Fibre and Whole grains	Website	https://www.heartandstroke.ca/healthy-living/healthy-eating/fibre-and-whole-grains	National
Nutrition/Diet	Fibre Facts	PDF handout	https://www.albertahealthservices.ca/assets/info/nutrition/if-nfs-fibre-facts.pdf	National
Nutrition/Diet	Free Health and Wellness Programs	PDF handout	https://cht.cdha.nshealth.ca/selectCourse.aspx?unitId=All#:~:text=The%20Community%20Health%20Teams%20in,available%20spaces%20are%20listed%20below	Nova Scotia
Nutrition/Diet	Grocery shopping the healthy way	PDF handout	https://www.albertahealthservices.ca/assets/info/nutrition/if-nfs-grocery-shopping.pdf	National
Nutrition/Diet	Half Your Plate	Website	https://www.halfyourplate.ca/	National
Nutrition/Diet	Hants Health and Wellness Team	Website	https://www.nshealth.ca/service-details/Hants%20Health%20and%20Wellness%20Team	Nova Scotia
Nutrition/Diet	Health Lifestyle Group	Website	https://www.nshealth.ca/service-details/Health%20Lifestyle%20Group	Nova Scotia
Nutrition/Diet	Healthy eating basics	Website	https://www.heartandstroke.ca/healthy-living/healthy-eating/healthy-eating-basics	National
Nutrition/Diet	Healthy Eating in Newfoundland & Labrador Resource Centre	Website	https://nlfoodaction.ca/for-your-health/	Newfoundland & Labrador
Nutrition/Diet	Healthy Snacking	PDF handout	https://www.albertahealthservices.ca/assets/info/nutrition/if-nfs-healthy-snacking.pdf	National
Nutrition/Diet	Label reading the healthy way	PDF handout	https://www.albertahealthservices.ca/assets/info/nutrition/if-nfs-label-reading.pdf	National
Nutrition/Diet	Meal-planning Toolkit	PDF handout	https://heartstrokeprod.azureedge.net/-/media/pdf-files/canada/meal-planning-toolkit/heart-and-stroke-meal-planning-toolkit.ashx?la=en&rev=28136383253f4fb28272b04b0ce488fc	National
Nutrition/Diet	Mediterranean Style of Eating	PDF handout	https://www.albertahealthservices.ca/assets/info/nutrition/if-nfs-mediterranean-style-of-eating.pdf	National
Nutrition/Diet	My Menu Planner	Online tool	https://www.unlockfood.ca/en/MenuPlanner.aspx	National
Nutrition/Diet	Nutrition and Lifestyle Choices to Manage Blood Pressure	PDF handout	https://albertahealthservices.ca/assets/info/nutrition/if-nfs-nutrition-and-lifestyle-choices-to-manage-bp.pdf	National
Nutrition/Diet	The DASH Diet to lower high blood pressure	Website	https://www.heartandstroke.ca/healthy-living/healthy-eating/dash-diet	National
Nutrition/Diet	The Mediterranean Diet: A Guide to Healthy Eating Eating	PDF handout	https://www.dietitians.ca/DietitiansOfCanada/media/Documents/Mediterranean%20Diet%20Toolkit/Mediterranean-Diet-Toolkit-A-Guide-to-Healthy-Eating-(handout).pdf	National
Nutrition/Diet	Tips to spend less money on food	PDF handout	https://www.albertahealthservices.ca/assets/info/nutrition/if-nfs-tips-to-spend-less-money-on-food.pdf	National
Nutrition/Diet	Using the Nutrition Facts Table: % Daily Value	PDF handout	https://www.canada.ca/content/dam/canada/health-canada/migration/healthy-canadians/alt/pdf/publications/eating-nutrition/label-etiquetage/fact-fiche-eng.pdf	National
Obesity	5As of Obesity Management—Practitioner Guide	Website	https://obesitycanada.ca/wp-content/uploads/2018/02/Practitioner_Guide_Personal_Use.pdf	National
Obesity	Adult Weight Management	Website	https://www.albertahealthservices.ca/info/Page13023.aspx	Alberta
Obesity	Edmonton Obesity Staging System	PDF handout	http://www.drsharma.ca/wp-content/uploads/edmonton-obesity-staging-system-staging-tool.pdf	National
Obesity	Obesity Canada website	Website	https://obesitycanada.ca	National
Obesity	Prevention and Harm Reduction of Obesity	PDF handout	https://obesitycanada.ca/wp-content/uploads/2021/05/4-Prevention-and-Harm-Reduction-v5-with-links.pdf	National
Obesity	Thinking about your weight? What about your health?—A healthy eating, active living and positive body image resource for adults	PDF handout	https://www.gov.nl.ca/hcs/files/publications-pdf-healthyliving-thinking-about-your-weight-what-about-health.pdf	National
Physical Activity	Canadian 24-Hour Movement Guidelines for Adults aged 18–64 years	Website	https://csepguidelines.ca/wp-content/uploads/2022/05/24HMovementGuidelines-Adults-18-64-ENG.pdf	National
Physical Activity	Canadian 24-Hour Movement Guidelines for Adults aged 65 years and older	Website	https://csepguidelines.ca/wp-content/uploads/2022/05/24HMovementGuidelines-Adults-65-ENG.pdf	National
Physical Activity	Move More, Sit Less	Website	https://cancer.ca/en/cancer-information/reduce-your-risk/move-more-sit-less	National
Physical Activity	Movement Counselling Tool for Adults aged 18–64 years	PDF handout	https://csepguidelines.ca/wp-content/uploads/2020/11/24MGCounsellingTool-Adults18-64_2-1.pdf	National
Physical Activity	Movement Counselling Tool for Adults aged 65 years and older	PDF handout	https://csepguidelines.ca/wp-content/uploads/2022/05/24MGCounsellingTool-Adults65.pdf	National
Physical Activity	Particpaction—At-home exercise videos	Website	https://www.participaction.com/en-ca/content/fit-break-videos	National
Physical Activity	Physical Activity for Adults (18 - 64 years)	Website	https://hi.easternhealth.ca/healthy-living/physical-activity/physical-activity-for-adults/	Newfoundland & Labrador
Physical Activity	Physical Activity for Older Adults (65 years +)	Website	https://hi.easternhealth.ca/healthy-living/physical-activity/physcial-activity-for-older-adults-65-years/	Newfoundland & Labrador
Physical Activity	Physical activity tips for adults (18–64 years)	PDF handout	https://www.canada.ca/content/dam/phac-aspc/migration/phac-aspc/hp-ps/hl-mvs/pa-ap/assets/pdfs/07paap-eng.pdf	National
Physical Activity	Physical activity tips for older adults (65 years and older)	PDF handout	https://www.canada.ca/content/dam/phac-aspc/migration/phac-aspc/hp-ps/hl-mvs/pa-ap/assets/pdfs/08paap-eng.pdf	National
Physical Activity	Resistance Exercises Handout	PDF handout	https://www.diabetes.ca/DiabetesCanadaWebsite/media/Managing-My-Diabetes/Tools%20and%20Resources/resistance-exercise.pdf?ext=.pdf	National
Physical Activity	Stay Active in the Winter	PDF handout	https://www.edmontonsouthsidepcn.ca/app/uploads/Stay-Active-in-Winter.pdf	National
Physical Activity	Walk this Way Kit	PDF handout	http://parc.ophea.net/sites/parc-dev.ophea.net/files/pdfs/Resources/WalkthisWayEnglishKit.pdf	National
Prostate Cancer	Benefits and Harms of PSA Screening	PDF handout	**EN:** http://canadiantaskforce.ca/wp-content/uploads/2016/12/CTFPHC_Prostate-Cancer_HarmsBenefits_FINAL.pdf **FR:** https://canadiantaskforce.ca/wp-content/uploads/2016/10/ctfphcprostate-cancerharmsbenefitsfrenchfinal.pdf	National
Prostate Cancer	Find prostate cancer	Website	https://cancer.ca/en/cancer-information/find-cancer-early/find-prostate-cancer	National
Prostate Cancer	Prostate Cancer Information	Website	https://myhealth.alberta.ca/health/pages/conditions.aspx?hwid=hw78220&lang=en-ca#hw78222	Alberta
Prostate Cancer	Prostate Cancer Resources (Cancer Care Ontario)	Website	https://www.cancercareontario.ca/en/types-of-cancer/prostate	Ontario
Prostate Cancer	Prostate Cancer Screening: Should I Have a PSA Test?	Website	https://myhealth.alberta.ca/health/pages/conditions.aspx?hwid=aa38144&lang=en-ca#zx3721	Alberta
Prostate Cancer	PSA Screening: Patient FAQ	PDF handout	**EN:** http://canadiantaskforce.ca/wp-content/uploads/2016/05/2014-prostate-cancer-patient-faq-colour-en.pdf **FR:** https://canadiantaskforce.ca/wp-content/uploads/2016/10/2014-prostate-cancer-patient-faq-french160111.pdf	National
Prostate Cancer	PSA Screening: Primary Care Practitioner FAQ	PDF handout	**EN:** https://canadiantaskforce.ca/wp-content/uploads/2016/05/2014-prostate-cancer-clinician-faq-colour-en.pdf **FR:** https://canadiantaskforce.ca/wp-content/uploads/2016/10/2014-prostate-cancer-clinician-faq-colour-fr.pdf	National
Prostate Cancer	PSA Test Decision Grid (Cancer Care Ontario)	PDF handout	https://www.cancercareontario.ca/sites/ccocancercare/files/assets/CCOProstateAntigen.pdf	Ontario
Prostate Cancer	Risks for prostate cancer	Website	https://cancer.ca/en/cancer-information/cancer-types/prostate/risks	National
Prostate Cancer	Should I get screened for prostate cancer?	PDF handout	**EN:** https://cdn.cancer.ca/-/media/files/cancer-information/resources/publications/should-i-get-screened-for-prostate-cancer/pcc_psa_brochure_2021_websingle_en.pdf **FR:** https://cdn.cancer.ca/-/media/files/cancer-information/resources/publications/should-i-get-screened-for-prostate-cancer/pcc_psa_brochure_2021_lowsingle_fr.pdf	National
Prostate Cancer	The harms of screening greatly outweigh the benefits	PDF handout	http://canadiantaskforce.ca/wp-content/uploads/2016/09/2014-prostate-cancer-infographic-en.pdf	National
Smoking/Tobacco Use	7 Tips to Lower Your Risk When Using Nicotine	PDF handout	https://www.nicotinedependenceclinic.com/en/Documents/Quick%20Tips.pdf	National
Smoking/Tobacco Use	AlbertaQuits	Program	https://albertaquits.healthiertogether.ca	Alberta
Smoking/Tobacco Use	Be Tobacco-Wise: Learn about the benefits of quitting smoking (First Nations)	PDF handout	**First Nations:** https://www.cancercareontario.ca/sites/ccocancercare/files/assets/TobaccoWiseBrochureFirstNations.pdf	Ontario
Smoking/Tobacco Use	Be Tobacco-Wise: Learn about the benefits of quitting smoking (Inuit)	PDF handout	**Inuit:** https://www.cancercareontario.ca/sites/ccocancercare/files/assets/TobaccoWiseBrochureInuit.pdf	Ontario
Smoking/Tobacco Use	Be Tobacco-Wise: Learn about the benefits of quitting smoking (Métis)	PDF handout	**Metis:** https://www.cancercareontario.ca/sites/ccocancercare/files/assets/TobaccoWiseFNIMBrochureMetis.pdf	Ontario
Smoking/Tobacco Use	Benefits of Quitting Smoking—Information for First Nations, Inuit, Métis and Urban Indigenous Peoples	PDF handout	https://tobaccowise.cancercareontario.ca/sites/cqco/files/assets/ITP-SmokingFlyer-BenefitsQuit_EN.pdf	Ontario
Smoking/Tobacco Use	Build Smoke-Free	Program	https://www.buildsmokefree.ca/en	National
Smoking/Tobacco Use	Build Your Quit Plan	Online tool	https://smokefree.gov/build-your-quit-plan	National
Smoking/Tobacco Use	Get help to quit smoking or vaping	Website	https://cancer.ca/en/living-with-cancer/how-we-can-help/get-help-to-quit-smoking	National
Smoking/Tobacco Use	One Step at a Time: Help Someone Quit	PDF handout	https://cancer.ca/en/cancer-information/resources/publications/osaat-help-someone-quit	National
Smoking/Tobacco Use	Help to Quit Tobacco	Website	https://www.smokershelp.net/	Newfoundland & Labrador
Smoking/Tobacco Use	Indigenous Tobacco Program	Website	https://tobaccowise.cancercareontario.ca/en	Ontario
Smoking/Tobacco Use	Live smoke-free	Website	https://cancer.ca/en/cancer-information/reduce-your-risk/live-smoke-free	National
Smoking/Tobacco Use	Lower-Risk Nicotine Use Guidelines	PDF handout	https://www.nicotinedependenceclinic.com/en/Documents/Recommendations.pdf	National
Smoking/Tobacco Use	My Quit: Your Personalized Quit Plan	Online tool	https://myquit.ca/prepare/	Ontario
Smoking/Tobacco Use	Quit Map: Find support to quit smoking and vaping nearby, online, by phone and elsewhere	Website	www.cancer.ca/quitmap	National
Smoking/Tobacco Use	Quitting Smoking (Tobacco)	Website	https://hi.easternhealth.ca/healthy-living/smoking-alcohol-cannabis/tobacco/	Newfoundland & Labrador
Smoking/Tobacco Use	Quitting smoking: Deciding to quit	Website	https://www.canada.ca/en/health-canada/services/smoking-tobacco/quit-smoking.html	National
Smoking/Tobacco Use	Quitting Tobacco Toolkit	Website	https://lunghealth.ca/tobacco/	National
Smoking/Tobacco Use	Smokers' Helpline	Program	http://www.smokershelpline.ca/	PEI, Ontario, Manitoba, Saskatchewan and Yukon
Smoking/Tobacco Use	Smoking and tobacco	Website	https://www.heartandstroke.ca/heart-disease/risk-and-prevention/lifestyle-risk-factors/smoking-and-tobacco	National
Smoking/Tobacco Use	Steps to Quitting Smoking—Information for First Nations, Inuit, Métis and Urban Indigenous Peoples	PDF handout	https://tobaccowise.cancercareontario.ca/sites/cqco/files/assets/ITP-SmokingFlyer-QuitSmoking_EN.pdf	Ontario
Smoking/Tobacco Use	STOP Smoking Cessation Program	Program	https://www.nicotinedependenceclinic.com/en/stop/join-stop	Ontario
Smoking/Tobacco Use	Talk Tobacco—Indigenous Quit Smoking and Vaping Support	Program	https://smokershelpline.ca/talktobacco	National
Smoking/Tobacco Use	Tobacco & Vaping Information for Health Professionals	Website	https://www.albertahealthservices.ca/info/Page17580.aspx	Alberta
Smoking/Tobacco Use	Tobacco and Oral Health	PDF handout	https://www.nshealth.ca/sites/nshealth.ca/files/patientinformation/1867.pdf	Nova Scotia
Smoking/Tobacco Use	Tobacco Free Nova Scotia	Program	https://tobaccofree.novascotia.ca/	Nova Scotia
Smoking/Tobacco Use	Tobacco, Vaping & Cannabis Program (Information for Albertans)	Website	https://www.albertahealthservices.ca/info/Page17590.aspx	Alberta
Smoking/Tobacco Use	One Step at a Time: You Can Quit	PDF handout	https://cancer.ca/en/cancer-information/resources/publications/osaat-you-can-quit	National
Social Services	211—National Resources and Supports	Website	http://211.ca/	National
Social Services	211 Resource Lists—Edmonton and Area	Online tool and PDF handout	http://edmonton.cmha.ca/211-resource-lists/#.WKdk0RBeYn1	Alberta
Substance Use	10 Steps to an ASSIST-Linked Brief Intervention	PDF handout	https://www.assistportal.com.au/download/10-steps-brief-intervention-2018/?wpdmdl=520&masterkey=5dd7d2646d566	National
Substance Use	Addiction & Mental Health Information for Health Professionals	Website	https://www.albertahealthservices.ca/info/Page11536.aspx	Alberta
Substance Use	Addiction Helpline	Website	https://www.albertahealthservices.ca/findhealth/Service.aspx?id=1008399&serviceAtFacilityID=1047128	Alberta
Substance Use	Addiction Services	Website	https://novascotia.ca/dhw/addictions/	Nova Scotia
Substance Use	Alcohol or drug use tools and programs	Website	https://mha.nshealth.ca/en/topics/symptoms/alcohol-or-drug-use	Nova Scotia
Substance Use	Alcohol, Smoking, and Substance Involvement Screening Test (ASSIST)—Lite	PDF handout	**PDF:** https://www.assistportal.com.au/download/assist-lite-form-download-for-printing/?wpdmdl=639&masterkey=5f43a151c1859 **Fillable Form:** https://www.assistportal.com.au/download/assist-lite-electronic-form/?wpdmdl=652&masterkey=602625689ff9c	National
Substance Use	Alcohol, Smoking, and Substance Involvement Screening Test (ASSIST) Tools and Resources	Website	https://www.assistportal.com.au/resources/	National
Substance Use	ASSIST Brief Intervention in Primary Care	Document	https://www.assistportal.com.au/download/assist-bi-manual-for-phc/?wpdmdl=780&masterkey60481d7d34624	National
Substance Use	Breaking Free: Confidential wellness and recovery support program	Online program	https://www.breakingfreeonline.ca/	Ontario and Newfoundland & Labrador
Substance Use	Fundamentals of Addiction	Website	https://www.camh.ca/en/professionals/treating-conditions-and-disorders/fundamentals-of-addiction	National
Substance Use	Get Help with Substance Use	Online tool	https://www.canada.ca/en/health-canada/services/substance-use/get-help-problematic-substance-use.html	National
Substance Use	Get help with substance use	Online tool	https://www.canada.ca/en/health-canada/services/substance-use/get-help-problematic-substance-use.html	National
Substance Use	Mental Health and Addictions Helplines and Resources	Website	https://www.gov.nl.ca/hcs/mentalhealth-committee/mentalhealth/	Newfoundland & Labrador
Substance Use	Older Adults (Where to go when you are looking for help)	PDF handout	https://www.camh.ca/-/media/files/community-resource-sheets/older-adults-resources-pdf	Ontario
Substance Use	Provincial Mental Health and Addictions Crisis Line	Website	https://mha.nshealth.ca/en	Nova Scotia
Substance Use	Stigma around drug use	Website	https://www.canada.ca/en/health-canada/services/opioids/stigma.html	National
Substance Use	Substance Use and Addiction	Website	https://hi.easternhealth.ca/healthy-living/mental-health/substance-use-and-addiction/	Newfoundland & Labrador
Substance Use	Substance Use and Withdrawal Management Services—Michael Garron Hospital	Program	https://www.tehn.ca/programs-services/mental-health-addiction/substance-use-withdrawal-management-services	Ontario
Substance Use	Supporting People Who Use Substances	PDF handout	https://www.heretohelp.bc.ca/sites/default/files/supporting-people-who-use-substances-update.pdf	National
Substance Use	Talking about drugs	Website	https://www.canada.ca/en/health-canada/services/substance-use/talking-about-drugs.html	National
Substance Use	Understanding Addiction	PDF handout	https://www.camh.ca/-/media/files/mi-index-other-languages/english-understanding-addiction	National
Substance Use	Wellness Together Canada	Program	https://www.wellnesstogether.ca/en-CA	National
Vaping/E-cigarettes	7 Tips to Lower Your Risk When Using Nicotine	PDF handout	https://www.nicotinedependenceclinic.com/en/Documents/Quick%20Tips.pdf	National
Vaping/E-cigarettes	About Vaping	Website	https://www.canada.ca/en/health-canada/services/smoking-tobacco/vaping.html	National
Vaping/E-cigarettes	E-cigarettes and Vaping	Website	https://www.nicotinedependenceclinic.com/en/electronic-nicotine-delivery-systems-(ends)	National
Vaping/E-cigarettes	E-cigarettes/Vaping Information and Resources	Website	https://smokershelp.net/vaping/	Newfoundland & Labrador
Vaping/E-cigarettes	Get help to quit smoking or vaping	Website	https://cancer.ca/en/living-with-cancer/how-we-can-help/get-help-to-quit-smoking	National
Vaping/E-cigarettes	Help to Quit Tobacco	Website	https://smokershelp.net/	Newfoundland & Labrador
Vaping/E-cigarettes	Lower-Risk Nicotine Use Guidelines	PDF handout	https://www.nicotinedependenceclinic.com/en/Documents/Recommendations.pdf	National
Vaping/E-cigarettes	My Quit: Your Personalized Quit Plan	Online tool	https://myquit.ca/prepare/	Ontario
Vaping/E-cigarettes	Quit Map: Find support to quit smoking and vaping nearby, online, by phone and elsewhere	Website	www.cancer.ca/quitmap	National
Vaping/E-cigarettes	Smoking and tobacco	Website	https://www.heartandstroke.ca/heart-disease/risk-and-prevention/lifestyle-risk-factors/smoking-and-tobacco	National
Vaping/E-cigarettes	Talk Tobacco—Indigenous Quit Smoking and Vaping Support	Program	https://smokershelpline.ca/talktobacco	National
Vaping/E-cigarettes	Tobacco & Vaping Information for Health Professionals	Website	https://www.albertahealthservices.ca/info/Page17580.aspx	Alberta
Vaping/E-cigarettes	Tobacco Harm Reduction—E-cigarettes	PDF handout	https://www.albertahealthservices.ca/assets/info/trp/if-trp-primer-tobacco-harm-reduction-e-cigarettes.pdf	Alberta
Vaping/E-cigarettes	Tobacco, Vaping & Cannabis Program Information for Albertans	Website	https://www.albertahealthservices.ca/info/Page17590.aspx	Alberta
Vaping/E-cigarettes	Vaping and quitting smoking	Website	https://www.canada.ca/en/health-canada/services/smoking-tobacco/vaping/smokers.html	National
Vaping/E-cigarettes	Vaping and Your Health	PDF handout	https://www.cancercareontario.ca/system/files_force/derivative/PCC_4159_Vaping_Handout_0.pdf?download=1	Ontario
Vaping/E-cigarettes	Vaping and Your Health brochure for First Nations, Inuit, Métis and Urban Indigenous Peoples	PDF handout	https://www.cancercareontario.ca/system/files_force/derivative/FNIM_VapingAndYourHealth_EN.pdf?download=1	Ontario
Vaping/E-cigarettes	Vaping Information and Resources	Website	https://hi.easternhealth.ca/healthy-living/smoking-alcohol-cannabis/vaping/	Newfoundland & Labrador
Vaping/E-cigarettes	Vaping Information and Resources	Website	https://novascotia.ca/vaping/	Nova Scotia
Vaping/E-cigarettes	Vaping Products Including E-cigarettes—Evidence summary (Ontario Health)	PDF handout	https://www.cancercareontario.ca/en/file/52376/download?token=sZvqxeF7	Ontario
Vaping/E-cigarettes	What you Need to Know About E-cigarettes	Website	https://cancer.ca/en/cancer-information/reduce-your-risk/live-smoke-free/what-you-need-to-know-about-e-cigarettes	National

*EN* English language, *FR* French language

### Updates to the BETTER toolkit

**Fig. 3:**
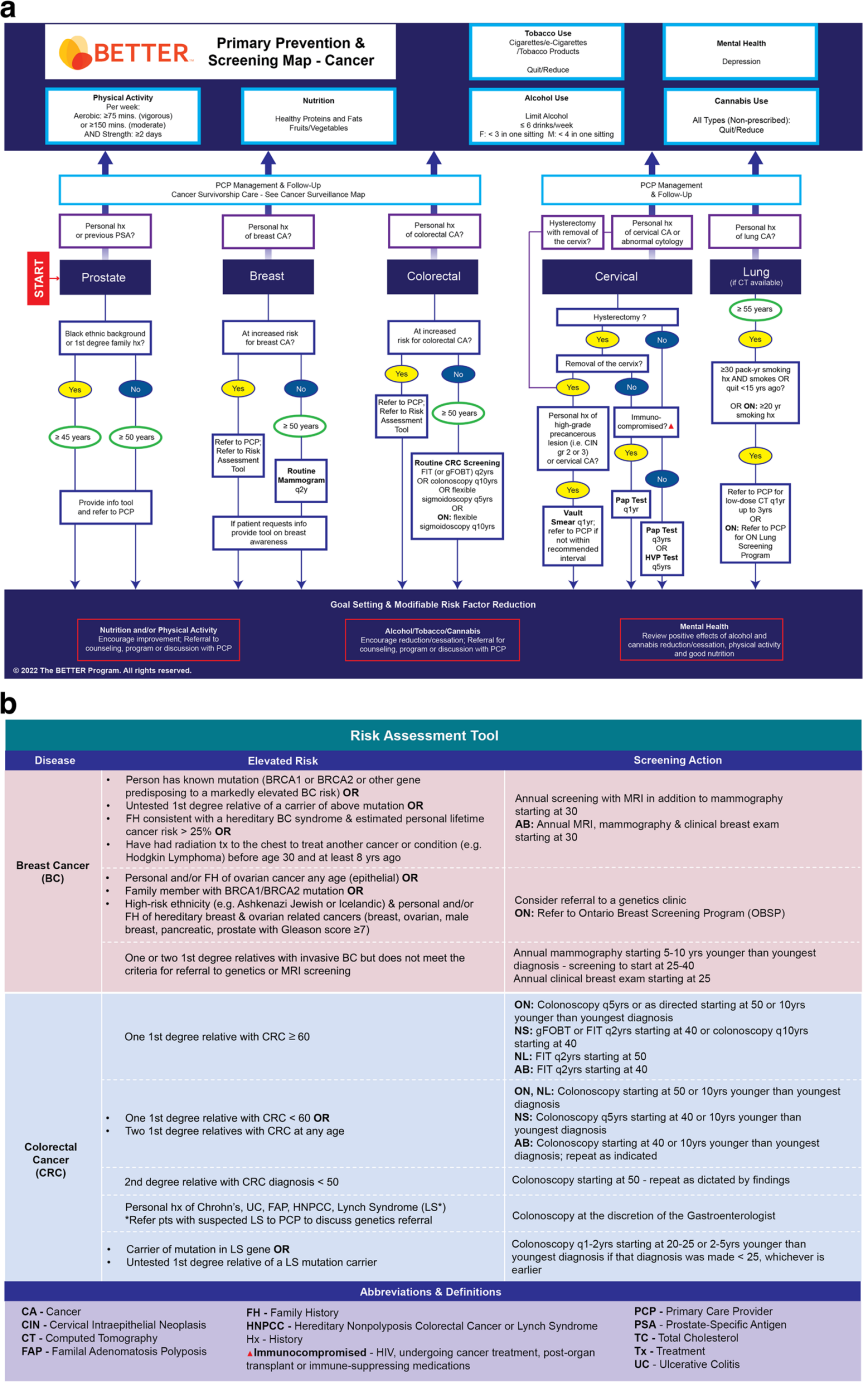
**a** The BETTER primary prevention and screening care map – cancer (front). **B** The BETTER Primary Prevention and Screening Care Map – Cancer (back)

**Fig. 4:**
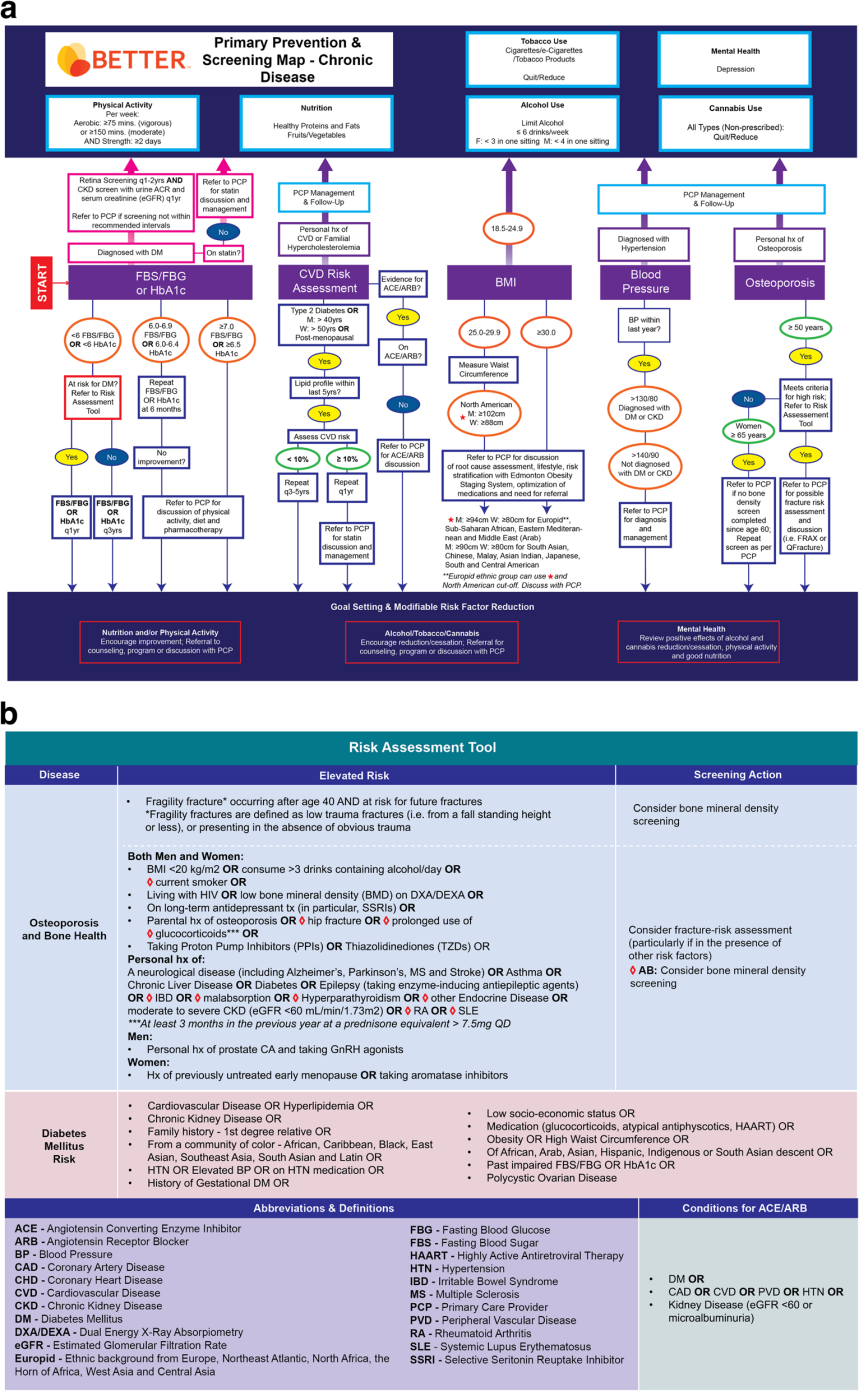
**a** The BETTER Primary Prevention and Screening Care Map – Chronic Disease (front). **B** The BETTER Primary Prevention and Screening Care Map – Chronic Disease (back)

**Fig. 5:**
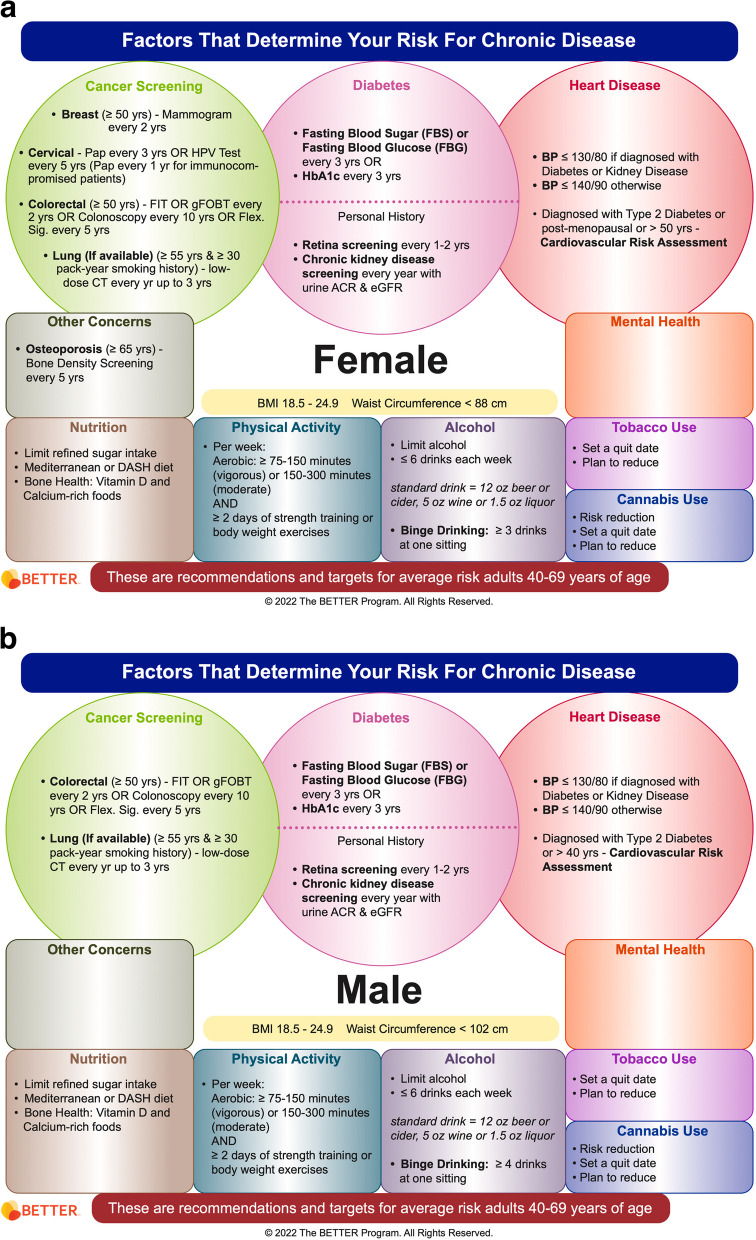
**a** The BETTER primary prevention bubble diagram: Male. **B** The BETTER Primary Prevention Bubble Diagram: Female

**Fig. 6:**
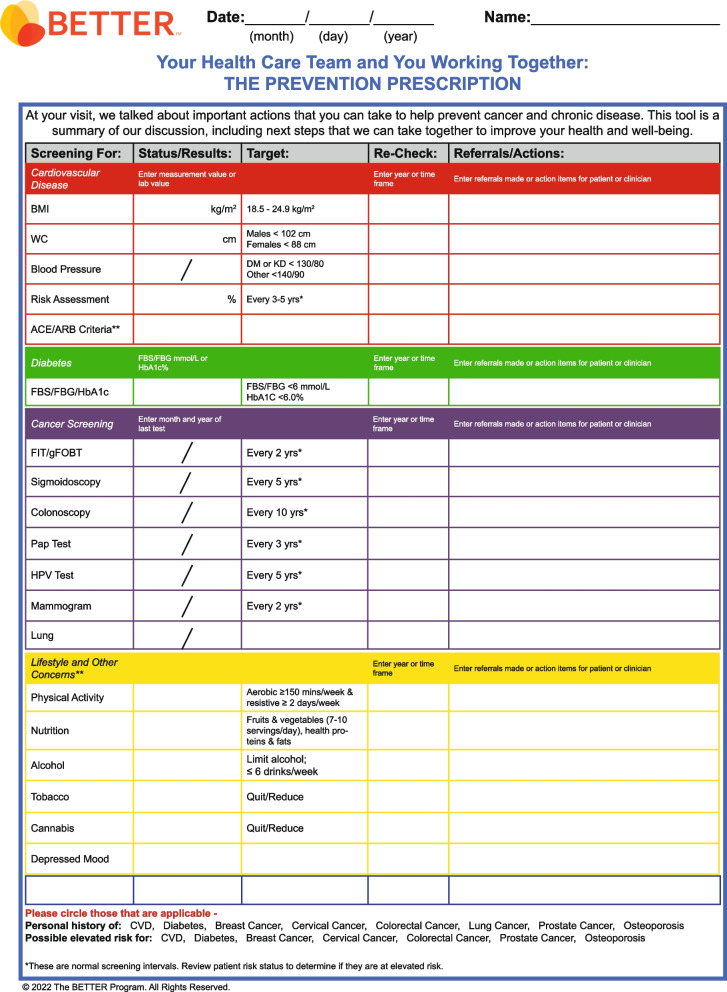
The BETTER prevention prescription

**Fig. 7:**
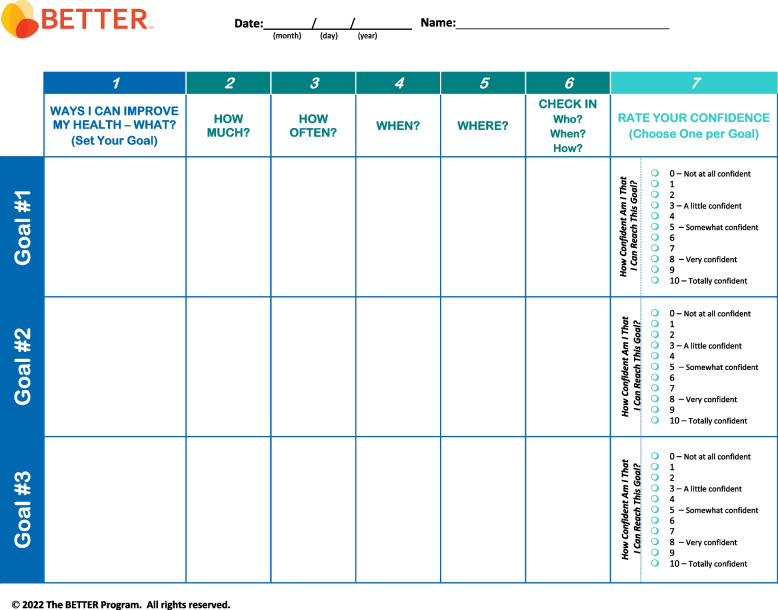
The BETTER goals sheet

The updated BETTER toolkit consists of clinical practice tools and resources that help assess patients, support patient education, shared decision-making, and self-management, including regional, provincial, and national resources that patients can access (via a provider or self-referral) to help them achieve their health goals. The BETTER toolkit includes:

1. The BETTER Primary Prevention and Screening Maps (Figs. 3a and b, 4a, and b), depict the harmonized clinical recommendations included in the BETTER program for adults 40–69 years of age (summarized in Table 2). Represented as care paths for each CCDPS topic, the maps are intended to help PCPs determine a patient’s eligibility for CCDPS as well as appropriate next steps, including frequency of screening and recommended screening modality based on personal medical history, family history, and genetics.
2. A patient survey, which captures a patient’s detailed prevention and screening history, including lifestyle risks and family history. The survey contains validated tools (e.g., PHQ-2, GPPAQ) and documents patients’ level of confidence and how prepared they are to make changes.
3. The BETTER Bubble Diagrams (Figs. 5a and b), which are patient- and clinician- friendly representations of the BETTER prevention and screening maps with sex specific targets for patients at average risk. A blank version can be used as a patient-teaching tool to illustrate the patient’s current health status and their risk factors for each ‘bubble’ to help guide the conversation.
4. A prevention prescription (Fig. 6), which provides a summary of the patient’s risk for cancer and chronic disease, their screening and prevention targets, and any follow-up actions that may be required.
5. A goals sheet (Fig. 7) that summarizes the patient’s personalized, self-directed, actionable S.M.A.R.T. goals.
6. A compilation of regional, provincial, and national resources and tools for patients and PCPs to support implementation of CCDPS recommendations, inform patients, and support patients’ health goals (Table 3).

## Discussion

Improving CCDPS in primary care is crucial to reducing the burden of cancer and chronic disease and increasing the sustainability of the healthcare system. In this paper, we describe the development of a comprehensive suite of resources and harmonization of recommendations that support CCDPS in patients 40–69 years of age. These are tailored to the individual by taking their personal medical history, family history, and genetics into consideration and for implementation in 4 Canadian provinces by incorporating provincial guidance. Stakeholders and end-users representing diverse perspectives (patients, policy makers, clinicians, and researchers) were engaged throughout the process to ensure that the clinical recommendations integrated into the BETTER program would be relevant, usable, and implementable.

Our evidence review involved a structured search of CPG databases, guideline developers, and grey literature to compile evidence-based clinical recommendations for CCDPS applicable to adults in the primary care setting. The resulting clinical recommendations expanded the age group for the BETTER program from 40–65 to 40–69 and added 3 new primary prevention and screening topics to the comprehensive scope of the program. In addition, clinical recommendations for secondary prevention of diabetes were introduced – retinopathy screening and screening for chronic kidney disease (see Table 2).

Informed by 51 international and Canadian CPGs and 22 guidelines from provincial organizations across 16 topics, the refined BETTER toolkit includes updated CCDPS care maps with succinct, clear, actionable recommendations that translate clinical evidence to PCPs to inform patient care, a streamlined patient survey to capture a patient’s prevention and screening history, and agenda setting tools to help set expectations for discussions with patients. The BETTER tools can be used at point of care to identify outstanding CCDPS actions and provide opportunities to address prevention and screening comprehensively, across many cancers and chronic diseases, with individual patients while considering their health goals, values, and preferences.

Initiated by the 1995 Institute of Medicine report *Setting Priorities for Clinical Practice Guidelines* [30], several decades of investment have resulted in robust methods to create high-quality guidelines; however, to achieve intended outcomes, CPGs must be implementable in real-world practice. The BETTER program has demonstrated that nuanced clinical tools can be designed to facilitate decision-making between PCPs and patients across multiple chronic diseases and lifestyle factors [2, 17, 18]. In this evidence review, we extended our topic scope, included health policy makers and patients in the evidence synthesis process, and tailored the included clinical recommendations for implementation in 4 Canadian provinces. The BETTER program process, grounded in the intersection between clinical practice, health policy, and systematic evidence, addresses a needed step to ensure feasible implementation of CPGs.

We recognize that our approach has limitations. Our population of interest was limited to adults 40–69 years of age with a focus on CCDPS and related risk factors. However, we believe that our approach may be useful to extend the work to different age groups, secondary prevention, and chronic disease management. All guidelines included in our review were published between 2016 and 2021 and as a result, recommendations from recent research, including guidelines published following the COVID-19 pandemic, would have been missed. For example, while the BETTER toolkit was being refined, two Canadian CPGs relating to cardiovascular disease [9] and alcohol use [31] were published in 2022 and 2023, respectively. To ensure that the recommendations used in the BETTER program remained clinically relevant, a subset of the BETTER CWG reviewed the CPGs and decided to include their recommendations. This highlights the importance of periodically and consistently reviewing the existing evidence to ensure that clinical practice is informed by the best current guidance. Though the clinical guidance was tailored to 4 jurisdictions in Canada and may not be applicable to other global jurisdictions or Canadian regions, the national recommendations included are relevant to a broad Canadian audience, and the tailoring methods used may prove useful to others when incorporating guidance for use in their context.

We developed a structured approach to synthesizing and blending CPG recommendations for application into primary care settings as described in our previous work [6, 11]. The process involved members of the Clinical Working Group sharing their diverse perspectives during group discussions to reach consensus for inclusion, harmonization, and synthesis of clinical recommendations extracted from high-quality CPGs. Though this approach is novel and not as recognized as other methodological approaches, such as the Delphi Method, it may still be used to guide and inform others on how to incorporate current clinical guidance into practice. Lastly, members of the Clinical Working Group were not asked to declare possible conflicts of interest prior to their involvement in the evidence review process; however, they represented diverse groups (PCPs, other healthcare professionals, patients, health policy specialists, content experts, researchers) from 4 Canadian Provinces, many of whom are authors on this manuscript and who have declared any competing interests here.

## Conclusions

The process used by the BETTER program to synthesize and harmonize international and Canadian CPG recommendations resulted in a suite of tools and resources to support CCDPS in primary care practice. This approach incorporates diverse perspectives of patients, PCPs, and health policy makers to ensure usability in real-world practice. Used together, the BETTER toolkit provides resources and tools that clearly and succinctly express the breath of high-quality clinical evidence that was synthesized into actionable recommendations to help inform patient care and enable primary care providers to address CCDPS comprehensively in their clinical settings. The methods used may be applicable to others contemplating integrating evidence across broad content areas in primary care to help facilitate comprehensive care. The updated BETTER toolkit is available to PCPs and interprofessional team members practicing in Canadian primary care settings through the BETTER program [12].

### Supplementary Information

**Additional file 1: Appendix 1.** Summary of Search Strategies.

**Additional file 2: Appendix 2. **Summary of Search Results.

**Additional file 3: Appendix 3. **Full AGREE II scores per clinical practice guideline (by topic area).

## Data Availability

Data sharing is not applicable to this article as no datasets were generated or analyzed as part of the work described in this manuscript.

## Abbreviations

AGREE II: Appraisal of Guidelines for Research & Evaluation II
BETTER: Building on Existing Tools to Improve Chronic Disease Prevention and Screening in Primary Care
BETTER WISE: Building on Existing Tools to Improve Cancer and Chronic Disease Prevention and Screening in Primary Care for Wellness of Cancer Survivors and Patients
CCDPS: Cancer and chronic disease prevention and screening
CCM: Chronic care model
C-CHANGE: Canadian Cardiovascular Harmonized National Guidelines Endeavour
CEP: Centre for Effective Practice
CIHR: Canadian Institutes of Health Research
COPD: Chronic obstructive pulmonary disease
COVID-19: Coronavirus disease of 2019
CPG: Clinical practice guideline
cRCT: Cluster randomized controlled trial
CT: Computed tomography
CWG: Clinical Working Group
GPPAQ: General Practice Physical Activity Questionnaire
PCP: Primary care provider
PHQ-2: Patient Health Questionnaire 2-item
PP: Prevention practitioner
SMART: Specific, measurable, attainable, realistic, time-bound
