# Supplementary material for: Harmonization of clinical practice guidelines for primary prevention and screening: actionable recommendations and resources for primary care
Source: BMC Prim Care. 2024 May 6;25:153. doi: 10.1186/s12875-024-02388-3 (PMC11071261; doi:10.1186/s12875-024-02388-3)
Supplement: Supplementary file 1 — Additional file 1: Appendix 1. Summary of Search Strategies. [file 12875_2024_2388_MOESM1_ESM.pdf]

Appendix 1 – Summary of Search Strategies

**Topic**	**Resource & Search Terms Used**									
	**Internet: Google**<br><br>**(First 3 pages of results reviewed for each search string)**	**Topic Specific**		**CPG Databases**		**Key CPG Developers**				
		**Ontario Health: Cancer Care Ontario**	**CPAC Cancer Guidelines Database**	**ECRI Guidelines Trust**	**CMA CPG Infobase**	**CTFPHC**	**USPSTF**	**NICE**	**SIGN**	**TOP Alberta**
**Cervical cancer screening**	- cervical cancer guideline<br>- cervical screening guideline<br>- cervical cancer screening guideline	Search limited to "guidelines and advice"<br>- cervical	Filter: type of cancer: Cervical	- cervical cancer<br>- cervical screen	- cervical cancer<br>- cervical screening	Manually reviewed by year of publication	Manually reviewed by current recommendations: Cancer	Manually reviewed by conditions and diseases: Cancer	Manually reviewed by list of current guidelines	Manually reviewed by cancer screening
**Colorectal cancer screening**	- colorectal cancer guideline<br>- colorectal screening guideline<br>- colorectal cancer screening guideline	Search limited to "guidelines and advice"<br>- colorectal	Filter: type of cancer: Colorectal	- colorectal cancer<br>- colorectal screen	- colorectal cancer<br>- colorectal screening	Manually reviewed by year of publication	Manually reviewed by current recommendations: Cancer	Manually reviewed by conditions and diseases: Cancer	Manually reviewed by list of current guidelines	Manually reviewed by cancer screening
**Prostate cancer screening**	- prostate cancer guideline<br>- prostate screening guideline<br>- prostate cancer screening guideline	Search limited to "guidelines and advice"<br>- prostate	Filter: type of cancer: Prostate	- prostate cancer<br>- prostate screen	- prostate cancer<br>- prostate screening	Manually reviewed by year of publication	Manually reviewed by current recommendations: Cancer	Manually reviewed by conditions and diseases: Cancer	Manually reviewed by list of current guidelines	Manually reviewed by cancer screening
**Breast cancer screening**	- breast cancer guideline<br>- breast screening guideline<br>- breast cancer screening guideline	Search limited to "guidelines and advice"<br>- breast	Filter: type of cancer: Breast	- breast cancer<br>- breast screen	- breast cancer<br>- breast screening	Manually reviewed by year of publication	Manually reviewed by current recommendations: Cancer	Manually reviewed by conditions and diseases: Cancer	Manually reviewed by list of current guidelines	Manually reviewed by cancer screening
**Lung cancer screening**	- lung cancer guideline<br>- lung screening guideline<br>- lung cancer screening guideline	Search limited to "guidelines and advice"<br>- lung	Filter: type of cancer: Lung	- lung cancer<br>- lung screen	- lung cancer<br>- lung screening	Manually reviewed by year of publication	Manually reviewed by current recommendations: Cancer	Manually reviewed by conditions and diseases: Cancer	Manually reviewed by list of current guidelines	Manually reviewed by cancer screening
**Type 2 diabetes**	- diabetes guideline<br>- type 2 diabetes guideline	n/a	n/a	- diabetes	- diabetes	Manually reviewed by year of publication	Manually reviewed by current recs: metabolic, nutritional, and endocrine conditions	Manually reviewed by conditions and diseases: Diabetes	Manually reviewed by list of current guidelines	Manually reviewed by endocrinology
**Cardiovascular disease**	- cardiovascular disease guideline<br>- cardiovascular guideline<br>- cardiovascular disease risk guideline	n/a	n/a	- cardiovascular	- cardiovascular	Manually reviewed by year of publication	Manually reviewed by current recs: Cardiovascular disorders	Manually reviewed by conditions and diseases: Cardiovascular conditions	Manually reviewed by list of current guidelines	Manually reviewed by cardiovascular

Topic	Resource & Search Terms Used									
	Internet: Google (First 3 pages of results reviewed for each search string)	Topic Specific		CPG Databases		Key CPG Developers				
		Ontario Health: Cancer Care Ontario	CPAC Cancer Guidelines Database	ECRI Guidelines Trust	CMA CPG Infobase	CTFPHC	USPSTF	NICE	SIGN	TOP Alberta
**Obesity**	- obesity guideline	n/a	n/a	- obesity	- obesity	Manually reviewed by year of publication	Manually reviewed by current recs: metabolic, nutritional, and endocrine conditions	Manually reviewed by conditions and diseases: Obesity	Manually reviewed by list of current guidelines	Manually reviewed by 'other' category
**Depression screening**	- depression guideline<br>- depression screening guideline<br>- depressive guideline<br>- mental health screening guideline	n/a	n/a	- depression	- depression	Manually reviewed by year of publication	Manually reviewed by current recs: mental health conditions and substance abuse	- depression	Manually reviewed by list of current guidelines	Manually reviewed by 'other' category
**Osteoporosis / bone density**	- osteoporosis guideline<br>- bone density guideline	n/a	n/a	- osteoporosis<br>- bone density	- osteoporosis<br>- bone density	Manually reviewed by year of publication	Manually reviewed by current recs: musculoskeletal disorders	-osteoporosis	Manually reviewed by list of current guidelines	Manually reviewed by 'other' category
**COPD**	- COPD guideline<br>- chronic obstructive pulmonary disease guideline	n/a	n/a	- COPD	- COPD	Manually reviewed by year of publication	- COPD	- COPD	Manually reviewed by list of current guidelines	Manually reviewed by 'other' category
**Hepatitis C**	- hepatitis C guideline<br>- hepatitis C screening guideline<br>- hepatitis guideline<br>- HCV screening guideline	n/a	n/a	- hepatitis C	- hepatitis	Manually reviewed by year of publication	Manually reviewed by current recs: infectious diseases	- hepatitis	Manually reviewed by list of current guidelines	Manually reviewed by 'infectious disease' category
**Screening for alcohol use**	- alcohol guideline<br>- alcohol screening guideline<br>- drinking guideline	n/a	n/a	- alcohol<br>- drinking	- alcohol<br>- drinking	Manually reviewed by year of publication	Manually reviewed by current recs: mental health conditions and substance abuse	- alcohol<br>- drinking	Manually reviewed by list of current guidelines	Manually reviewed by 'other' category
**Screening for tobacco use**	- tobacco guideline<br>- tobacco screening guideline<br>- smoking guideline<br>- smoking screening guideline	n/a	n/a	- tobacco<br>- smoking	- tobacco<br>- smoking	Manually reviewed by year of publication	Manually reviewed by current recs: mental health conditions and substance abuse	- tobacco<br>- smoking	Manually reviewed by list of current guidelines	Manually reviewed by 'other' category

Topic	Resource & Search Terms Used									
	Internet: Google (First 3 pages of results reviewed for each search string)	Topic Specific		CPG Databases		Key CPG Developers				
		Ontario Health: Cancer Care Ontario	CPAC Cancer Guidelines Database	ECRI Guidelines Trust	CMA CPG Infobase	CTFPHC	USPSTF	NICE	SIGN	TOP Alberta
**Screening for diet**	- healthy eating guideline<br>- healthy eating clinical practice guideline<br>- diet clinical practice guideline	n/a	n/a	- healthy eating<br>- diet	- healthy eating<br>- diet	Manually reviewed by year of publication	- healthy eating<br>- diet	- healthy eating<br>- diet	Manually reviewed by list of current guidelines	Manually reviewed by 'other' category
**Screening for physical activity**	- physical activity guideline<br>- exercise guideline	n/a	n/a	- physical activity	- physical activity	Manually reviewed by year of publication	- physical activity	- physical activity	Manually reviewed by list of current guidelines	Manually reviewed by 'other' category
**Screening for vaping / e-cigarette use**	- vaping guideline<br>- e-cigarette guideline	n/a	n/a	- vaping<br>- e-cigarette	- vaping<br>- e-cigarette	Manually reviewed by year of publication	Manually reviewed by current recs: mental health conditions and substance abuse	- vaping<br>- e-cigarette	Manually reviewed by list of current guidelines	Manually reviewed by 'other' category
**Screening for cannabis use**	- cannabis screening guideline<br>- cannabis guideline<br>- marijuana screening guideline<br>- marijuana guideline	n/a	n/a	- cannabis<br>- marijuana	- cannabis<br>- marijuana	Manually reviewed by year of publication	Manually reviewed by current recs: mental health conditions and substance abuse	- cannabis<br>- marijuana	Manually reviewed by list of current guidelines	Manually reviewed by 'other' category
**Screening for drug use**	- drug use guideline<br>- substance use guideline	n/a	n/a	- drug use<br>- substance use	- drug use<br>- substance use	Manually reviewed by year of publication	Manually reviewed by current recs: mental health conditions and substance abuse	- drug<br>- substance	Manually reviewed by list of current guidelines	Manually reviewed by 'other' category
**Screening for lifestyle factors in primary care (general search)**	- chronic disease prevention guideline<br>- preventive screening guideline<br>- preventive health guideline<br>- lifestyle disease guideline<br>- lifestyle screening guideline	n/a	n/a	- lifestyle	- lifestyle	Manually reviewed by year of publication	- screening	Manually reviewed by lifestyle and wellbeing	Manually reviewed by list of current guidelines	Manually reviewed by 'other' category

CPAC = Canadian Partnership Against Cancer; CMA CPG = Canadian Medical Association Clinical Practice Guidelines Infobase; CTFPHC = Canadian Task Force on Preventive Health Care; NICE = National Institute for Health and Care Excellence; SIGN = Scottish Intercollegiate Guidelines Network; TOP = Toward Optimized Practice; and USPSTF = U.S. Preventive Services Task Force.

Appendix 2 – Summary of Search Results

Topic	Phase 1: Literature Search											Phase 2: Full Text Review and Initial Quality Appraisal	Phase 3: Full AGREE II Appraisal - Final Included CPGs	Additional CPGs with Relevant Recs Identified in Other Topics	Total CPGs Included Per Topic *Note: Some CPGs included in more than one topic*
	Internet	Topic-Specific		CPG Databases		Key CPG Developers					Phase 1: Search Results Title/Abstract Review				
		OH: CCO	CPAC CGD	ECRI	CMA CPG	CTFPHC	USPSTF	NICE	SIGN	TOP Alberta					
**Cervical cancer screening**	90	18	27	51	15	13	15	24	21	4	278	6	2		**2**
**Colorectal cancer screening**	90	47	86	41	27	13	15	24	21	4	368	31	5		**5**
**Prostate cancer screening**	90	47	56	40	30	13	15	24	21	4	340	18	3		**3**
**Breast cancer screening**	90	23	87	93	35	13	15	24	21	4	405	17	3		**3**
**Lung cancer screening**	90	62	67	64	26	13	15	24	21	4	386	28	2		**2**
**Type 2 diabetes**	60	n/a	n/a	133	76	13	23	11	21	11	348	15	4		**4**
**Cardiovascular disease**	90	n/a	n/a	120	21	13	22	13	21	1	301	16	3		**3**
**Obesity**	30	n/a	n/a	98	61	13	23	11	21	16	273	5	3		**3**
**Depression screening**	120	n/a	n/a	82	21	13	16	3	21	16	292	8	0		**0**
**Osteoporosis / bone density**	60	n/a	n/a	43	19	13	5	3	21	16	180	14	6		**6**
**COPD**	60	n/a	n/a	30	18	13	102	10	21	2	256	10	3		**3**
**Hepatitis C**	120	n/a	n/a	27	25	13	22	19	21	11	258	10	4		**4**
**Screening for alcohol use**	90	n/a	n/a	75	22	13	12	2	21	16	251	7	3	2	**5**
**Screening for tobacco use**	120	n/a	n/a	120	22	13	12	8	21	16	332	12	2	4	**6**
**Screening for diet**	90	n/a	n/a	91	19	13	131	4	21	16	385	6	1	6	**7**
**Screening for physical activity**	60	n/a	n/a	138	11	13	102	1	21	16	362	6	3	6	**9**
**Screening for vaping / e-cigarette use**	60	n/a	n/a	10	4	13	12	0	21	16	136	10	0		**0**
**Screening for cannabis use**	120	n/a	n/a	12	21	13	12	1	21	16	216	10	1		**1**
**Screening for drug use**	60	n/a	n/a	44	48	13	12	11	21	16	225	11	3		**3**
**Screening for lifestyle factors in primary care (supplemental search)**	150	n/a	n/a	93	12	13	129	12	21	16	446	3	0	n/a	n/a
**Total**	1740	197	323	1405	533	260	710	229	420	221	6038	243	51		

CMA CPG = Canadian Medical Association Clinical Practice Guidelines Infobase; CPAC CGD = Canadian Partnership Against Cancer's Cancer Guideline Database; CTFPHC = Canadian Task Force on Preventive Health Care; NICE = National Institute for Health and Care Excellence; OH: CCO = Ontario Health: Cancer Care Ontario; SIGN = Scottish Intercollegiate Guidelines Network; TOP = Toward Optimized Practice; and USPSTF = U.S. Preventive Services Task Force.
