# Supplementary material for: Harmonization of clinical practice guidelines for primary prevention and screening: actionable recommendations and resources for primary care
Source: BMC Prim Care. 2024 May 6;25:153. doi: 10.1186/s12875-024-02388-3 (PMC11071261; doi:10.1186/s12875-024-02388-3)
Supplement: Supplementary file 2 — Additional file 2: Appendix 2. Summary of Search Results. [file 12875_2024_2388_MOESM2_ESM.pdf]

Appendix 2 – Summary of Search Results

Topic	Phase 1: Literature Search											Phase 2: Full Text Review and Initial Quality Appraisal	Phase 3: Full AGREE II Appraisal - Final Included CPGs	Additional CPGs with Relevant Recs Identified in Other Topics	Total CPGs Included Per Topic *Note: Some CPGs included in more than one topic*
	Internet	Topic-Specific		CPG Databases		Key CPG Developers					Phase 1: Search Results Title/Abstract Review				
		OH: CCO	CPAC CGD	ECRI	CMA CPG	CTFPHC	USPSTF	NICE	SIGN	TOP Alberta					
**Cervical cancer screening**	90	18	27	51	15	13	15	24	21	4	278	6	2		**2**
**Colorectal cancer screening**	90	47	86	41	27	13	15	24	21	4	368	31	5		**5**
**Prostate cancer screening**	90	47	56	40	30	13	15	24	21	4	340	18	3		**3**
**Breast cancer screening**	90	23	87	93	35	13	15	24	21	4	405	17	3		**3**
**Lung cancer screening**	90	62	67	64	26	13	15	24	21	4	386	28	2		**2**
**Type 2 diabetes**	60	n/a	n/a	133	76	13	23	11	21	11	348	15	4		**4**
**Cardiovascular disease**	90	n/a	n/a	120	21	13	22	13	21	1	301	16	3		**3**
**Obesity**	30	n/a	n/a	98	61	13	23	11	21	16	273	5	3		**3**
**Depression screening**	120	n/a	n/a	82	21	13	16	3	21	16	292	8	0		**0**
**Osteoporosis / bone density**	60	n/a	n/a	43	19	13	5	3	21	16	180	14	6		**6**
**COPD**	60	n/a	n/a	30	18	13	102	10	21	2	256	10	3		**3**
**Hepatitis C**	120	n/a	n/a	27	25	13	22	19	21	11	258	10	4		**4**
**Screening for alcohol use**	90	n/a	n/a	75	22	13	12	2	21	16	251	7	3	2	**5**
**Screening for tobacco use**	120	n/a	n/a	120	22	13	12	8	21	16	332	12	2	4	**6**
**Screening for diet**	90	n/a	n/a	91	19	13	131	4	21	16	385	6	1	6	**7**
**Screening for physical activity**	60	n/a	n/a	138	11	13	102	1	21	16	362	6	3	6	**9**
**Screening for vaping / e-cigarette use**	60	n/a	n/a	10	4	13	12	0	21	16	136	10	0		**0**
**Screening for cannabis use**	120	n/a	n/a	12	21	13	12	1	21	16	216	10	1		**1**
**Screening for drug use**	60	n/a	n/a	44	48	13	12	11	21	16	225	11	3		**3**
**Screening for lifestyle factors in primary care (supplemental search)**	150	n/a	n/a	93	12	13	129	12	21	16	446	3	0	n/a	n/a
**Total**	1740	197	323	1405	533	260	710	229	420	221	6038	243	51		

CMA CPG = Canadian Medical Association Clinical Practice Guidelines Infobase; CPAC CGD = Canadian Partnership Against Cancer's Cancer Guideline Database; CTFPHC = Canadian Task Force on Preventive Health Care; NICE = National Institute for Health and Care Excellence; OH: CCO = Ontario Health: Cancer Care Ontario; SIGN = Scottish Intercollegiate Guidelines Network; TOP = Toward Optimized Practice; and USPSTF = U.S. Preventive Services Task Force.
